# Simulated patients and their reality: An inquiry into theory and method

**DOI:** 10.1016/j.socscimed.2021.114571

**Published:** 2022-05

**Authors:** Veena Das, Benjamin Daniels, Ada Kwan, Vaibhav Saria, Ranendra Das, Madhukar Pai, Jishnu Das

**Affiliations:** a Department of Anthropology, Johns Hopkins University, Baltimore, USA; b Georgetown University, Washington DC, USA; c Department of Medicine, University of California at San Francisco, San Francisco, USA; d Department of Gender, Sexuality, and Women's Studies, Simon Fraser University, Burnaby, Canada; e Institute for Socio-Economic Research on Development and Democracy, Delhi, India; f McGill International TB Centre, McGill University, Montreal, Canada; g Manipal McGill Centre for Infectious Diseases, Manipal Academy of Higher Education, Manipal, India; h Center for Policy Research, New Delhi, India

**Keywords:** Simulated standardized patients, Epistemology, Quality of care, Health care providers, Health markets

## Abstract

Simulated standardized patients (SSP) have emerged as close to a ‘gold standard’ for measuring the quality of clinical care. This method resolves problems of patient mix across healthcare providers and allows care to be benchmarked against preexisting standards. Nevertheless, SSPs are not real patients. How, then, should data from SSPs be considered relative to clinical observations with ‘real’ patients in a given health system?

Here, we reject the proposition that SSPs are direct substitutes for real patients and that the validity of SSP studies therefore relies on their ability to imitate real patients. Instead, we argue that the success of the SSP methodology lies in its counterfactual *manipulations* of the possibilities available to real careseekers – especially those paths not taken up by them – through which real responses can be elicited from real providers.

Using results from a unique pilot study where SSPs returned to providers for follow-ups when asked, we demonstrate that the SSP method works well to elicit responses from the provider through conditional manipulations of SSP behavior. At the same time, observational methods are better suited to understand what choices real people make, and how these can affect the direction of diagnosis and treatment. A combination of SSP and observational methods can thus help parse out how quality of care emerges for the “patient” as a shared history between care-seeking individuals and care providers.

## Highlights

• We assess how simulated standardized patients (SSPs) compare to ‘real’ patients.
• SSPs elicit real responses from providers for different counterfactual patients.
• However, SSPs are not informative of observed variation in patient behavior.
• SSPs illustrate how providers behave with different types of patients.
• Observation studies can complement SSPs by studying patient responses.

## 1 Introduction

There is an increasing consensus among health researchers and policymakers that Universal Health Coverage implies not only better access to healthcare, but better access to *quality* healthcare (Das et al., 2018; Kruk et al., 2018). In India, concerns over quality arose in early studies that demonstrated the widespread and indiscriminate use of drugs (Greenhalgh, 1987; Kamat and Nichter, 1998; Eck and Harper, 2011). They were later refined using pathway studies for conditions like tuberculosis (TB), which revealed considerable diagnostic delays leading to adverse public health consequences and the development of drug-resistant strains (Sreeramareddy et al., 2014).

An unsolved problem in these studies was that, if patients were not diagnosed correctly to begin with, adjudicating the quality of care they received required multiple ancillary assumptions: Researchers had no basis to compare what the patient received relative to what they actually needed. The fact that medical records were frequently missing or nonexistent only added to the problem as retrospective chart reviews, which are frequently used (but still problematic) to measure quality in high-income contexts, were not feasible (Gilbert et al., 1996). Studies attempted to circumvent this problem by implementing knowledge tests, but the discovery of systematic differences between knowledge and practice suggested that such methods were poor proxies for clinical quality (Das et al., 2008; Rethans et al., 1991).

While originally conceptualised as a set of distinct 'building blocks' (World Health Organization, 2007), later iterations of health systems frameworks emphasized dynamic interaction across components (Van Olmen et al., 2012, Zwama et al., 2021) including health infrastructure, available facilities, provider competence and networks of laboratories, all of which affect provider decisions and patient care. Here, the clinical interaction is not simply a unilateral application of provider knowledge, but is often a negotiated practice in which system-wide effects influenced patient behavior and provider decisions. The recent literature around “patient-centric” systems extends this idea more generally (Davis, 2017), but such conceptions continue to be limited by the lack of data on whether a patient was treated correctly for their disease given the health system structure.

A breakthrough that has successfully addressed this problem, we propose, is the use of simulated standardized patients (SSPs) in population-based samples. SSP studies have shown that the question – “*was the patient correctly managed for their underlying condition*” – can be accurately and reliably measured. The first population-based SSP studies expanded their use from pedagogical tool to a field method for measuring and monitoring quality (Das et al., 2012). Since then, SSPs have been used in multiple contexts, developing and expanding research protocols as learning has accumulated about their use for population-based measures of quality.

In an SSP study, locally recruited individuals are trained to portray pre-specified conditions using a standardized script with contextually and biomedically appropriate symptom descriptions. SSP studies seek to understand how providers diagnose and treat patients and inform quality improvement. As the results are easy to convey and the method allows researchers to compare the care that was given *in relation to the underlying illness*, SSPs have emerged as a flexible tool that can provide critical data on the quality of clinical care, as opposed to provider knowledge. The use of SSPs addresses well-known problems of quality measurements, from confounding due to patient- and case-mix to Hawthorne effects, whereby providers change their behavior when they are observed (Leonard and Masatu, 2006). SSP studies have documented widespread inappropriate care, ranging from misdiagnosis to unnecessary diagnostic tests and medications (Kwan et al., 2019).

Despite their expanding use, one recurring concern is that SSPs are not real patients and therefore the extent to which we should privilege or discount data from SSPs relative to direct clinical observation remains unclear (Aujla, 2020). In SSP studies, the presentation is standardized and this is what allows for valid comparisons across different providers, presentations, or other populations. However, in real life, patients show enormous variation with regard to symptom presentation and behavior in the clinical setting. For instance, patients differ in how passive or active they are in questioning the provider about the required tests or treatments; they also make decisions as consumers around the trustworthiness of recommendations based on local knowledge about financial interests of providers in local laboratories and pharmacy shops (Das, 2015, Mol, 2008, Saria,). This variation among real patients forces us to reassess the kind of information that can be extracted about provider behavior through the SSP method relative to observations of clinical encounters with real patients.

We discuss the nature of this reassessment, showing that the methodology raises difficult epistemological issues relating to theory and method. Specifically, we ask what is entailed in patient simulation in terms of the ontology of the patient and the epistemology of simulation. By the ontology of a patient we mean, what a “patient” is within a clinical interaction, and how this identity is generated, taking into account the reality of an individual care-seeker (e.g., features of the person as a social persona), a care provider's perception of that person, and the shared narrative of disease and care as it emerges during the clinical interaction(s) between the two in the context of the whole health system. This motivates our core concern, which is what we can learn about the care of “real” patients by observing an individual simulating such an identity and a provider responding to them.

We argue that it is tempting – but too simplistic – to view SSPs as merely good or bad imitations of real patients. Instead of assuming that increased realism in SSP design would improve the validity of the method, we believe that concerns about realism must be examined within the context of a specific question and health system. We further argue that one key role of SSP studies is the construction of counterfactual manipulations of the simulation, which can provide critical insights on provider behavior within a milieu as well as across different contexts.

Building on our research on TB in urban India (Kwan et al., 2018), we ground these ideas within an empirical exploration of gains in accuracy through the use of “follow-up” SSP visits, which is one oft-suggested approach to increasing realism. The specific problem is that existing SSP studies are based on a single visit to a provider because of the logistical difficulties of following up if asked to. If the accurate measurement of provider behavior requires the development of a shared experience of the pathway to care between patient and provider, single visits miss the fact that recommendations given by providers may evolve as the interaction advances (Armstrong, 1998; Das, 2015).

To understand how repeat visits contribute to greater realism, we report descriptive results from a pilot study in which SSPs returned to providers in multiple follow-ups. Surprisingly, our evidence suggests that providers rarely unilaterally advance patient care across such visits, but that active prompting by or negotiation with the patient may do so. We discuss how this (tentative) finding of the role of active prompting *by the patient* takes us away from the comparison with real patients, while simultaneously elevating the importance of SSPs as a pragmatic tool for improving quality of care.

At the heart of this seeming contradiction is the observation that our pilot study increases our confidence that the standard SSP method, with a one-time visit, remains a valid measure of care *if* patients signaled full compliance and did not negotiate with providers over their recommendations. However, we know little about how provider behavior would change in response to the behaviors of “real” patients, particularly when the treatment is not leading to improvement necessitating a potential change in how patients communicate with the provider. The subtle point we make is that, for some questions, *that knowledge may not be necessary*.

Our first point, related to quality-improvement strategies, is that even if we do not know how real patients behave in these circumstances, we can still understand how *providers* would behave under different counterfactuals. That is, we could assess (with a larger experiment) if providers would be more likely to converge to the correct diagnosis *if* repeat patients were to prompt them in certain ways. This has the practical implication that a patient-education campaign on communication in repeat visits could improve quality of care. It parallels the idea in a road safety study, where researchers put posters in vans used for public transport in Kenya, asking travelers to ‘speak up’ if the driver was driving rashly (Habyarimana and Jack, 2011). As the researchers demonstrated, travelers seldom objected without the stimuli, but the new *affordance* led them to speak up, reducing accidents and fatalities. The point is not whether travelers generally spoke up in the face of rash driving (they don't), but whether a change in circumstances could lead them to do so and therefore change the behavior of drivers.

Our second point is that, even if the evolution of treatment involves patient-provider negotiation and the construction of a shared memory of interactions, it is possible to construct counterfactual levers on these branches of possibility in a standard single-visit design. A broad range of case histories and personal backgrounds are available to simulate any particular disease, mapping the progression of the disease to different case scenarios that represent the disease in early and later stages, or in mild versus more severe manifestation. For instance, one SSP may report that they ‘have had a cough for the last three weeks’, while a second may report that they ‘have had a cough for the last three weeks *that has not improved despite seeking medical care*.’ If doctors behave the same way regardless of whether that initial visit was with the same doctor, we will have replicated a repeat design using single visits.

This line of reasoning ultimately leads to a finer partitioning of the questions that SSP data are well equipped to answer. For questions regarding the care trajectories various providers are likely to take compared against each other or even against themselves under alternative case presentations, the standardization and simulation are crucial. There are other questions that are better answered with observational methods. For instance, if we are trying to understand how patient inputs bring out the negotiated character of diagnosis and treatment, ethnographic methods with direct clinical observation may be more informative.

Thus, it is best to consider SSPs and real patients as two different configurations of possibilities, rather than one being real and the other being its imitation. The SSP method works well to elicit responses from providers, but collapses at the point where a key decision is required to be made by patients. The method allows us to understand hypothetical – and, critically, *counterfactual* – possibilities under the control of the researcher through “*if*-*then*” statements, such as, *if* the SSP returns to the provider having done what the doctor asked of them, *then* the doctor would do X. It is, by design, silent on the plausibility of the *if* statement. Through simulation of these different configurations of possibilities, the SSP method can become an important tool to understand provider behavior both in terms of patient behavior (passive versus active patients) and in terms of the information they give about things like finances, family support, or the likelihood of switching providers. Ultimately, such modelling might be a useful next step to take into the design of SSP studies, rather than attempts to make SSPs more realistic, which will always be limited by the fundamental question: Realistic in relation to which patients?

## 2 Simulation as method: A conceptual framework

Questions around the validity of simulation are not unique to healthcare. In fact, the use of simulations has a storied history in artificial intelligence. Unlike computer-based simulations in AI and psychology, however, where machine activity is evaluated in terms of how closely it can mimic human actions, SSPs are not simply intelligent agents functioning in a virtual environment. In terms of perception and experience, they are as real as the providers with whom they interact.

The asymmetry that simulation introduces is an asymmetry of information in that SSPs (unlike real patients) already know which disease they are representing, and the information they give providers is standardized. It is not influenced by such considerations as real constraints on their ability to comply or their ability to recall the history of symptoms or other social constraints, unless such constraints are deliberately introduced as part of the simulation. This difference between SSPs and real patients is essential to the design of SSP studies.

The goal here is not to accurately portray real patients in all aspects, but to elicit provider responses within clinical interactions that are real *from the provider's point of view*, even though responses to questions given by SSP are part of the simulation. As Floyd (2015, 2017) argues, the philosophical grounding of simulation models is not about comparing the real and the simulated, but how one might read “possibility through actuality” into discussions of the real. We extend this to propose that the question of realism in SSP studies cannot be framed as Turing-type tests that judge the value of the simulation by similarity to any real patient. In fact, SSPs pass such tests with flying colors—when researchers have returned to participating providers and asked them whether they recognized any SSPs, less than 5% do so (Das et al., 2015; Boffa et al., 2021).

More helpful than Turing-type tests are the models that have been used in geographical models of wayfinding. The vocabulary developed there will be instrumental to understanding SSP studies, and we offer an example due Raubal (2001a), in which the information-seeking behavior of travelers in an airport as they attempt to reach their departure gate is modeled as a simulation. A conceptual vocabulary helps distinguish the same agents and objects as “real” for some purposes and “simulated” for others.

The first concept is that of “cognizing agents”, defined by Anshakov and Gergely (2010) as “*either a living entity (particularly a human being), or a group of them or a technical system, which can adapt to the changing conditions of the external environment”*. In an airport, for example, Raubal (2001b) argues travelers and staff are “cognizing agents”. The second concept is that of “non-cognizing objects”: ticket counters, signage boards giving directions, or other objects that make up the conceptual domain of wayfinding behavior. Objects are further divided in this model into “*bona fide”* objects with natural boundaries (such as architecture external to the simulation) and “*fiat*” objects with boundaries created by human decisions, like waiting areas, security zones, and signage in the airport.

Each object offers several *affordances* to the cognizing agents, which “*comes from ecological psychology; [affordances are] what an object, an assemblage of objects, or an environment enables people to do*” (Raubal, 2001b). Affordances might be contrasted with constraints; affordances reside neither in the agents nor the objects but arise from interactions among them. Affordances allow multiple possible uses, each of which may or may not be intended or desired by the designer. Which of these will be taken up by a traveler depends on their past experiences and their understandings of the various objects; a seasoned traveler might navigate differently from a first-time flyer.

The conceptual vocabulary of wayfinding models therefore distinguishes real people going about their normal life from the domain-specific ontology of the same persons when seen as cognitive agents placed in a specific environment in which different objects allow certain affordances. Such a view allows us to understand the value of observing a real person in a simulation constructed for the practical aim of discovering, for instance, design problems in that environment. The purpose of simulation in this space is *not* to reproduce the factual patterns of behavior taken up by actual travelers, but to understand how their behavior might be altered if the space were to be reconfigured — to explore counterfactual possibilities in pursuit of a better understanding of the experience of specific agents of interest. One can see that such work might be used to design better airports; we suggest this idea extends to the simulation of patients. Specifically, methodically exploring how providers and patients take up counterfactual affordances of the health system in various carefully-designed simulated patient interactions might allow the system to be reconfigured to improve care.

## 3 Patient simulation, standardization, and realism

We now situate SSP research in this framework from the premise that a person performing as a simulated patient elicits the same responses from health care providers as a real patient would, an assumption supported by the low detection rates in SSP studies. From there, we need to understand how the experiences of the two might relate beyond one being an imitation of another. That is, we need to understand how experimental variation of simulation parameters can allow us to learn about the way real providers and patients *might* take up the various affordances of the health system in ways that naturalistic observation cannot.

There is a faithful mapping of some aspects of the empirical world into patient simulations, while in others the “patient” is an entity within the domain created through simulation alone, for instance, through *if*-*then* statements. We can learn, for instance, that *if* a patient says that they have “had a cough for 4 weeks”, or visits a pulmonologist, *then* they will receive a specific type of care (say, a sputum test). By altering these *if* statements in specific ways, we can systematically understand provider responses and discover affordances that could improve care for real patients. For instance, in our TB work we discovered that doctors would often not provide a free voucher for tests. SSPs were then used to assess *if* asking for the voucher increased provision; if yes, a next step may be to put up posters informing patients of their entitlement, an affordance that some patients might adopt and some providers may respond to, both of which can be recovered through appropriate experimental design.

The cognizing agents, then, in the SSP simulation are both the healthcare providers and the SSPs (Brenner, 2009). There is an asymmetry in the self-knowledge of these two: Providers are wholly real and believe they are treating a real patient, but SSPs know that they are not the people they claim to be and they know what disease the simulated person suffers from. This asymmetry implies the interaction may be understood as real for some purposes – the course the interaction takes is directed by the provider and cannot be anticipated or controlled. By contrast, patient behavior, such as responses to questions, are standardized based on biomedical laws of disease progression in addition to being designed as *if*-*then* statements controlling the scenario conveyed to providers.

The domain-specific objects in the SSP case are both those generated in the real world and those simulated for the purpose of a case presentation. Simulated objects are primarily medical records, such as a chest X-ray or a lab report documenting the presence of TB. These documents are, in one sense, real: They are generated in an actual laboratory on real film or paper, and there is a real person whose chest cavity has been imaged. But all the names are false, and the dates are continuously updated to correspond to the visits of the SSPs. These objects are thus “*bona fide*” with natural boundaries, but they are also “*fiat*” objects that have been created and given meaning by decisions of the study team to meet domain-specific needs. The important point is that the referent of these reports is the SSP: There is no real patient or radiologist to whom the report can be traced.

Finally, the existence of the SSPs in the clinic and the objects they carry constitute the affordances. Providers can take advantage of the SSP's presence to ask questions, conduct examinations, and ask for test results. These affordances will be taken up to different degrees. As a concrete example, in cases where SSPs carry chest X-rays or sputum tests, only 13% of providers asked to see the X-ray and 19% asked to see the sputum test (Kwan et al., 2018).

To stay within the vocabulary of cognizing agents, *bona fide* objects, and fiat objects, we therefore propose four characterizations of SSPs:

1. Providers are real cognizing agents who behave as they would with real patients.
2. SSPs, although they are trained to give standardized responses to potential questions, are cognizing agents in that they must interpret the questions and requests of the provider in the clinic in real time. However, to the extent that their simulated responses are placed in the clinical environment to elicit responses from providers, they are simultaneously fiat objects.
3. The *bona fide* objects are the space of the clinic and the instruments used in the clinic such as stethoscopes, medicines, injections, and the like.
4. Other objects such as X-rays and lab reports referring to SSPs during the interaction are additionally fiat objects, in that human intentions have altered their *bona fide* status.

The crucial consideration is whether the SSP method produces data on provider decisions usable for comparing the quality of care for TB in various potential scenarios. Our interest in SSPs does not come exclusively from the fact that SSPs can be made to appear like real patients, though this factor is important to ensure that providers think they are engaged with real patients. The importance of the SSP method lies in its ability to elicit and observe responses from the providers that are *unambiguously* real and can be acted upon, such as making pedagogic interventions to motivate providers towards generating appropriate diagnostic tests. The interesting aspect of SSPs is therefore not that they are “not real” patients, but that the simulation elicits real responses from providers, a study feature that depends on the accuracy of the simulation in both the medical and the social domain.

In the medical domain, all answers to medically-relevant questions posed by the provider can be anchored to natural laws in the trajectory of the disease and are standardized. For instance, the symptoms and history questions (if asked) are answered in identical ways across providers and across SSPs.

A crucial additional component that goes beyond the elements constructed to interact with the medical domain is the social persona of the SSP, which must be constructed in the genre of realism, so that the providers are led to treat SSPs *as if they were* real patients. This second component of the SSP script entails social knowledge, such as the readiness to improvise during the interaction according to the context in which the provider is located. If the provider asks the SSP a question about social markers such as caste, or about their neighborhood, the SSP must be prepared to respond accurately and believably, even though this may be tangential to the strict medical definitions of the scenario.

Social knowledge is often deeply connected to the fashion in which a real patient in a given milieu would present themselves: Real patients do not walk into clinics and describe symptoms from a textbook. There is an element of realism in the way a symptom is described that creates non-arbitrary relations between symptoms and disease. As one example, when describing difficulties in breathing, asthma patients in the communities we work in often use phrases such as “*uppar ki saans upar, neeche ki saans neeche*”, translating as, “the top breath remains stuck on the top, the bottom one on the bottom”. However, TB patients describe their symptoms differently, saying, for instance that they have difficulty breathing (*saans ki takleef hai*) or that they get breathless (*saans phool jata hai*). SSPs presenting with TB were taught to avoid the phraseology appropriate to asthma because it could mislead the provider.

The standardization of responses in this way performs four functions: (a) it uses expressions frequently used in the community to represent symptoms and experience; (b) it presents the information to the provider needed to suggest a specific diagnosis in the most direct possible way; (c) it ensures that the character of the SSP is carefully controlled and; (d) in the case of patients representing later stages of the disease, it informs the provider that the patient was not treatment-naive, but had switched providers or followed some other history of prior care that would be relevant for the complaint. This is also why SSPs play a key role in script development: They flesh out their biographies with appropriate social markers such as personal and caste names, likely occupations, and relative readiness to offer a personal history to a provider if asked.

Finally, providers are chosen according to a sample design that captures heterogeneity in terms of training and location. The providers are not simulated, so their selection has implications for the external generalizability and statistical power of such a study. We liken this process to the use of data generated through wayfinders’ interaction with real airline terminals and agents: The data in the SSP studies is generated through their interactions with real providers in the health system. SSP studies essentially have an empirical agenda: Did the structure of the simulation create the conditions of possibility under which relevant data for understanding and intervening on specific goals (such as reducing time to diagnosis) could be observed?

## 4 Returning to the scene: Follow-up SSP visits

Notwithstanding the general argument that the realism of SSPs is secondary to their scientific utility — that we are interested in comparing specific possibilities rather than generalizing across all possibilities — it is important that we accurately observe and measure the real relevant behaviors of the provider in those situations. We therefore turn now to a specific concern about realism in “single-visit” SSP studies. Since providers often ask patients to return if their condition does not improve, it is possible they would narrow down the patient's condition to the correct diagnosis after failing to do so on the first visit.

We describe a pilot study using such “follow-ups” of SSPs to the same provider, extending the patient simulation past the initial interaction. This procedure has not been attempted before and the results have important implications for thinking about real patients as both empirical agents and as constructs, as well as understanding new possibilities and limitations of the SSP method. We explore (a) whether providers are likely to move care forward in an unprompted fashion in subsequent interactions with the same patient, and (b) whether case histories that involve prior treatment by other providers are believable and valid.

In our previous work, we used multiple SSP scenarios to evaluate provider decisions about case management at different stages of disease progression (Das et al., 2015). The basic scenario was that of an undiagnosed person with TB presenting with two weeks of cough and fever. Subsequent scenarios presented more affordances, marking a progression of the disease. For instance, in the second scenario the SSP has (purportedly) consulted with another provider, been given antibiotics without a diagnosis, and carries a chest X-ray that (if examined) indicates abnormalities suggestive of TB. In a third scenario, the SSP had (purportedly) visited a government hospital and carries a microbiological smear test report that (if examined) indicates the presence of TB. The fourth scenario is intended to simulate a multi-drug-resistant TB infection. The individual reports having received a TB diagnosis the prior year and starting a course of anti-TB treatment, but discontinuing medication when their symptoms improved. They report experiencing recurrent symptoms.

To take the data from the three advanced scenarios as generally valid would require that the provider believed the case histories as described by the SSPs, even though (a) they had never actually happened; and (b) even if they had, they had not happened *under that provider's care*. The degree to which these particular “simulated histories” are real or believable – and the degree to which other histories could be simulated in other cases – will inform the kinds of questions future SSP studies might ask and answer.

Our study highlighted that the number of providers who diagnosed TB increased when presented with either the chest X-ray or the microscopy report (Kwan et al., 2018). Further, the first scenario of a person presenting with two weeks’ cough yielded considerable variation in diagnostic pathways. This is a crucial point that marks the start of our investigation with these “follow-up” visits.

Our pilot study addresses two challenges. First, we asked how much knowledge of future interactions was missed in any of these single-visit SSP interactions and, second, what value did the advanced cases offer when presented to the same providers without personal experience of the case history. For example, providers who failed to order an appropriate diagnostic test the first time they saw a new patient might do so in subsequent interactions.

In January 2018, we undertook a pilot study to test these “follow-up” visits with a small sample of 16 providers from three different localities in Delhi, of which 8 were AYUSH (those trained in alternative streams of medicine, particularly Ayurveda, Unani, and Homeopathy) and 8 held degrees in biomedicine, reflecting the diversity of qualifications in the city (Das, 2015, Das et al., 2020). We retained only the first case scenario of the person with undiagnosed TB since the other possibilities are designed around progression from this point.

SSPs began the initial interaction with the standard opening statement: “*Doctor, I have had a cough since some time which is not getting better and some fever too*.” They would answer provider questions regarding history, symptoms, and prior diagnostic tests according to the original SSP protocol. Then, if they were asked to return for a second consultation, they would comply. The SSP would return exactly when the provider had requested, pretend to have taken medications if they had been given any, and report that there was no improvement in their symptoms. The SSP behavior in this case is similar to the behavior of some real patients who might either register lack of improvement because they were being inappropriately treated or, even if appropriately treated, they expected to feel better much sooner. As the specific relationship between the patient and the provider evolved, it required “de-standardizing” the simulations. Each interaction took a unique path, as no two providers offered the same trajectory of care. The forking of a single initial case scenario into such multiple possibilities required active decisions about the path of the simulation to be made by the research team – within the structure of *if*-*then* responses in real time.

Critically, to implement the follow-up study, our research team also had to determine at which point in each series of interactions the follow-up visits should be terminated if the provider continuously failed to reach the conclusion that the patient potentially had TB. What, after all, should a provider think of a patient who they are not able to cure, but who continues to come back to them repeatedly? Initially, the plan was that if, at any stage, the provider correctly diagnosed TB or asked for a TB-related test, this would be regarded as correct case management. If the provider did not ask for a TB-related test by the third visit, the interaction would be terminated; if they did, the SSP would return with the X-ray or other test results to see how the provider would react (in effect, folding the advanced case scenarios into the natural progression of the basic case).

However, after the first two visits by most of the SSPs were completed, the team realized that most providers continued asking very few questions, and that the actual clinical interactions still only lasted a few minutes. More surprisingly, some providers appeared to have no recall of previous visits by the SSP, asking who they were and why they had come (even though the previous visit occurred two or three days before). Even in those second interactions, patients were often dispensed the same medicines but with an addition of another medicine to be bought from a pharmacist, and were typically asked to return back in the following few days – a finding consistent with our large-scale study, but nevertheless surprising to see repeated even on the second encounter with the providers.

The team then decided that if providers continued to simply dispense medicines and ask the patient to return again after two visits, SSPs would proactively ask on the third visit: “*Doctor, the medicines are not making the cough any better – is there anything to worry about?*” This “trigger” statement was introduced for all SSPs going forward, except one who had already completed three rounds of visits at the time this decision was made. Therefore, three *if* statements were introduced in the research, which potentially distinguish these SSPs from a generic “real” patient:

1. The SSP will fully comply if the provider demands something, such as to buy medicines or produce a chest X-ray if asked to obtain one (subject to the safety limitations in typical SSP protocols).
2. The SSP will return to the provider at the specified time and report not having experienced a feeling of improvement through the treatment interaction.
3. At a specific juncture of the evolving interactions, the SSP will express a worry about not feeling better.

In reality, among real patients, full compliance and follow-up may be low or intermittent. Administrative data from Mumbai suggested that when an AYUSH provider gave a free X-ray voucher, some patients chose not to redeem it for up to 6 months. Further, patients may choose to switch to another provider rather than return to the same provider if the treatment does not help. We observe that the trigger statement seemed to induce providers who had not generated a TB test or referral (from both the AYUSH and biomedical groups) to offer some alternate diagnosis, such as that of “sore throat” or “allergic cough” to reassure the patient about the correctness of their chosen treatment protocol.

## 5 Results of the follow-up study: Trajectories of care

To begin, we report a positive methodological result from the pilot study. Follow-up visits by SSPs are feasible and they do not appear to increase the risk of detection of the SSP by the providers. None of our interactions resulted in suspicion by the provider that the patient was not real, and no adverse events occurred at any time. All the visits were successfully completed and documented. As the study was not designed to provide a statistical analysis of the evolution of care across follow-up visits, we include all SSP case narratives in the online Appendix.

While the results are positive in terms of feasibility, implementing the SSP methodology with follow-up visits required intensive, real-time (re-)development of scripts. Even though all interactions started in an identical fashion, no two took the same direction. Each had to be re-scripted by the team after each interaction to prepare for the next, which also had to be scheduled according to the provider's direction. If such a study were done with a larger number of providers, any given initial presentation would quickly evolve into a large number of disparate scenarios which have to be individually managed.

**Fig. 1:**
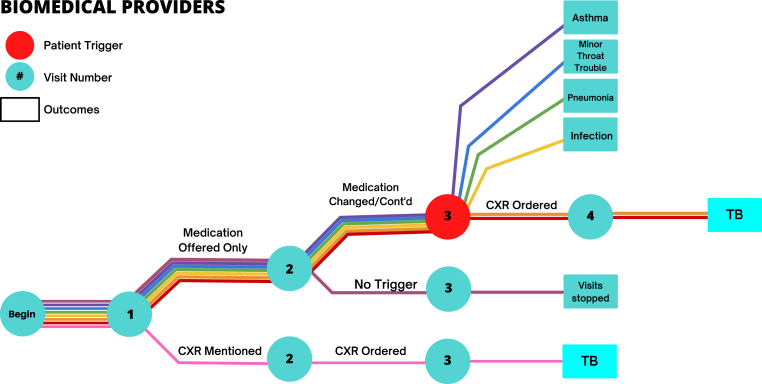
Trajectories of care among AYUSH practitioners. Note: This figure illustrates the care patterns given among the eight AYUSH practitioners visited by standardized patients as a part of the pilot study. Each circle indicates a visit to the provider; the number in the circle indicates the visit number. The red circle indicates patients who gave the trigger statement to the provider in that interaction. The lines indicate the eight patient-provider interactions, with each color representing a single pairing. Boxes indicate outcomes where the interaction was deemed complete by the study team and further follow-up visits were stopped for that patient. TB = tuberculosis; CXR = chest X-ray.

**Fig. 2:**
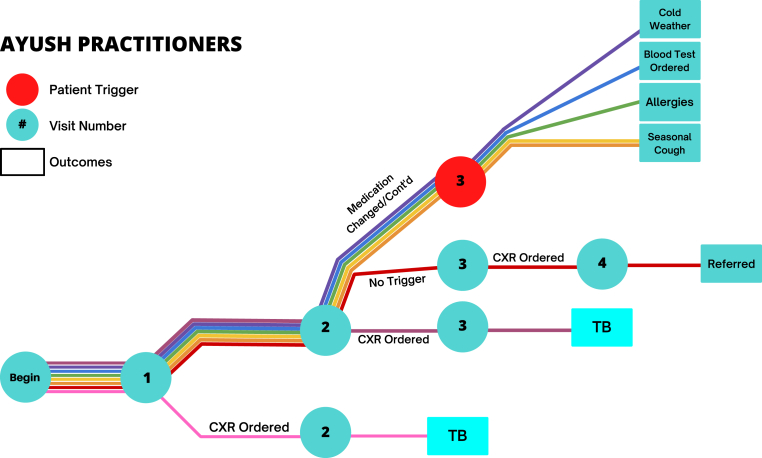
Trajectories of care among biomedical practitioners. Note: This figure illustrates the care patterns given among the eight biomedical practitioners visited by standardized patients as a part of the pilot study. Each circle indicates a visit to the provider; the number in the circle indicates the visit number. The red circle indicates patients who gave the trigger statement to the provider in that interaction. The lines indicate the eight patient-provider interactions, with each color representing a single pairing. Boxes indicate outcomes where the interaction was deemed complete by the study team and further follow-up visits were stopped for that patient. TB = tuberculosis; CXR = chest X-ray.

Fig. 1 and Fig. 2 illustrate the care paths followed by each provider across several interactions, including how providers behaved after the trigger statement. Among the AYUSH providers, one ordered a chest X-ray on the first visit and diagnosed TB; one did so on the second visit; and one did so on the third. Of these, two then referred the patient to either a government hospital or a private chest physician. For the remaining five, the SSPs were instructed to introduce the trigger query on the third interaction; all five were given an alternative diagnosis in response to the query.

Among biomedically-trained providers, one provider mentioned the possibility of a chest X-ray on the first visit and proceeded to order it on the second. One provider ended visits before the trigger statement was fully implemented. None of the other six providers mentioned any TB testing on the first or second visit, but instead gave medication on the first visit and continued to so on the second. On the third visit to these providers, SSPs were instructed to add the trigger query: In response to this, four of the providers offered an alternative diagnosis. Two moved forward to order a chest X-ray after the trigger query, and both diagnosed TB based on the X-ray when the SSP returned with it.

We cannot draw conclusions about what specific information the trigger query might have communicated to providers. Among the possibilities are that the patient is concerned about the seriousness of her condition; or that failing improvement or a new strategy on the part of the provider, the patient might switch to another provider. In fact, two AYUSH providers – who presumably anticipated such a switch – already had the connections in their networks with labs or specialized clinics and recommended another provider to the SSPs.

In light of these observations, there are three conclusions from these pilot results. First, the results suggest that single-visit SSP study designs do not typically mischaracterize the direction that a given interaction will take if left to the provider's discretion across the follow-up visits they often request. It appears that providers will not converge towards correct management by trial and error unless they initially suspect the true cause. In only two of our sixteen cases did a provider meaningfully advance care toward a chest X-ray without additional prompting from the SSP if the provider had not initially requested or mentioned it, although we cannot ascertain the causality of the trigger within this study.

Second, the pilot results additionally suggest that using advanced SSP scenarios with more extensive histories – even without a prior history with the provider – is a functional approach to understanding cascades of care. Providers seemed to anticipate the possibility of the patient seeking care elsewhere, and they behaved broadly similarly with SSPs under their own care as they had with SSPs reporting prior care from other providers in other studies. Namely, when SSPs in this study presented the chest X-ray in follow-up visits with the same providers, they were treated similarly to SSPs in other studies who presented it initially: They were either initiated into treatment protocols (by biomedical providers) or referred to higher level care (by AYUSH providers).

Third, the results suggest that in this context some form of active negotiation, prompting, or provider-switching is required from the patient to move towards “more serious” care. If this finding is validated in a larger experimental SSP study, it suggests that information campaigns that lead patients to express greater concern about their condition may trigger faster diagnoses.

In sum, the additional realism offered by following provider instructions for future visits does not appear to offer substantial new information about the appropriateness of care beyond that obtained through single-visit SSP approaches. However, it links patient to provider behavior in a way that can potentially be used to improve the quality of care.

## 6 Discussion: Health systems, medical knowledge, multiple realities

Recent discussions in medical anthropology have gone beyond earlier preoccupations with the idea of disease as a social construct that would bracket the biological reality of the disease and instead shifted attention to questions arising partly from the ontological turn in anthropology and partly from the shift in thinking of health systems as not simply static contexts of clinical interactions but dynamic possibilities that offer patients and providers different kinds of affordances. As a result of this shift, anthropologists do not take for granted that what happens in clinical interactions is a straightforward application of medical knowledge by the provider to the management of disease, with the patient conceived as ‘compliant’ or ‘non-compliant’ (Liberati et al., 2015).

Willems (1992) notes that, in many studies of clinical interactions, the patient is taken to be all “lay” and the provider to be all “expert”. Positing a more complex relation between what the doctor knows and what the patient knows, Willems illustrates this point with a case study of a patient reporting with chronic conditions of breathlessness. He says the knowledge of the technical instruments is a distributed knowledge — the doctor knows more about what to look for in an X-ray, but the patient may know more about how to use a peak flow meter. Yet the issues are not limited to the knowledge of medical technologies alone — as we saw, there is suggestive evidence that the provider is cued to the hints about the patient's willingness to comply, the likelihood of being able to retain the patient, as well as how well the provider is networked with laboratories, to arrive at a decision of obtaining a diagnostic test (Hunt and Arar, 2001).

In this context, we submit that the SSP method provides an important intervention that presents new ways of understanding provider competence behavior, especially in a milieu where the heterogeneity in provider quality necessitates such an evaluation. If, as Mol (2002) argues, what providers do in a clinical setting is not exclusively an application of what they know cognitively, but also how they respond to patient triggers, then how does our study contribute to the understanding of provider behavior as a series of health system enactments?

Mol's ethnography of the way a disease moves in a hospital setting from the clinic to the laboratory and back to the clinic, demonstrates how medicine attunes to and shapes its objects; it is not, she claims, about how medicine knows its objects, but how it shapes it. Her examples of the different ways that medical objects are constructed in the clinic versus the laboratory, and what kind of negotiations lead to decisions about the diagnosis and treatment, radically alters the idea that medical knowledge is about representation. Instead, as she rightly says, disease is better seen as enactments or practices, and it is only when intricacies of enactment are bracketed that disease becomes a single entity located within the body of the patient.

In Mol's discussions, however, “the patient” appears primarily at the moments in which a medical decision has to be made in the light of different evaluations from the laboratory and the clinic or in the differences between two providers. As one vascular surgeon says during a discussion with her: “*But of course we always talk with the patient first. And we only operate if that patient has severe complaints. And they must be pretty motivated too, before we operate*.” (p.94) Mol takes this kind of reference to patients to claim that medicine does not silence its object (the patient) as many claim, but rather that it constitutes the patient himself or herself as multiple. Because Mol's study is located in a well-functioning hospital, she can herself bracket certain questions regarding how to judge different practices that go on to constitute diagnosis and treatment: “*If the objects of medicine are enacted in a variety of ways, truthfulness is no longer good enough. Somehow, questions need to be asked about the appropriateness of various enactments of the body multiple and its diseases. I don't ask such questions here*.” (p.182)

In contrast to Mol's statements about judgment, we have no choice but to ask how provider responses are to be judged as appropriate or not, and more importantly, how might we act on them. This perspective motivates our extensive construction of the physical and social history of the SSP and of the trajectory of TB from less severe to more severe cases with a view towards learning about the different configurations of possibilities that provider behavior reveals in each case. It also inspires the pilot investigation reported here as a lens to view the relationship between these scripts and the progression of care with a given provider over time. Going further, the character of this multiplicity is the essential epistemological question behind whether or not researchers can successfully investigate care cascades and complex case histories using only single-visit SSP studies in which no real history or relation exists outside the scripted simulation.

What we have presented through the SSP method is the varying configurations of possibilities that are often ironed out of ethnographic accounts of both provider and patient behavior, because of the need for what Cartwright calls “prepared descriptions” to rewrite observed actions and events in more abstract ways to fit the framework of a model or theory (Cartwright and McMullin, 1984; Toon, 2010). In those accounts, we end up with a figure of a “typical patient in this milieu”, with a bracketing of shifts in behavior by providers and patients in the course of the disease – what Canguilhem (2012) called the continuous generation of new norms.

These norms are shaped as much by the different components of the health system as by the experiences specific individual patients have. We submit that the SSP method provides an opening to interrogate the negotiated possibilities providers and patients have, given specific system-level affordances and constraints. By configuring alternative counterfactual possibilities, the method can alert us to where failures of diagnosis and treatment protocols may occur, and thus the various points in the system at which interventions may be enacted to provide better health care. Conceptualizing the resources of the health system in terms of an assemblage of affordances and constraints, rather than in terms of other strict divisions like “hardware” and “software”, allows us to think of providers and patients as acting within the health system – shaping it as well as being shaped by it through the everyday decisions they make as they navigate these structures.

Let us, as conclusion, listen to the words of a provider in Patna (Saria, 2020). The provider is trying to explain to the patient the constraints within which they both work: He asks that the patient get a second chest X-ray because the quality of the X-ray they had obtained was poor and grainy, and the provider could not decipher whether there were patches on the lung. The patient's husband was in a quarrelsome mood: “*Why did you not send us to a better lab right at the start? Why did you make us waste our money on this one*?”

The provider responded with his own frustrations. “*So, what should I have done? Send you to Delhi to get an X-ray where the machines are worth 1 crore rupees? If you want, I can just make you spend your money unceasingly.*” This is the kind of exchange that cannot be captured in the *if*-*then* structure of SSP statements, because all possible patient reactions cannot be anticipated in general. This statement points to frustration with the facilities, the difficulties of affording good quality care, and other constraints of the health system within which providers and patients work. Yet it is just one possibility, among others, of how this specific clinical encounter might have shaped up – what we are really interested in here is what other outcomes may have been possible for this patient. This is the approach from which SSP study designs are most fruitfully developed: Exploring the range of possibilities available to a single individual within a given health system. That approach does not require thinking that other real patients provide a standard against which the value of such simulated patients can be measured.

We hope that continued exploration of the relation of SSPs to real patients draws attention to these difficult problems in the location and multiplicity of the patient-provider interactions and the different affordances of the health system. In addition, we hope that by more fully understanding the possibilities that can be explored from that perspective, new questions can be asked and answered through simulation which can be of help to health systems in reducing delays in diagnosis and promoting adherence to correct treatment.

## CRediT author statement

**Veena Das:** Conceptualization, methodology, analysis, resources, writing, supervision, administration, investigation, funding acquisition. **Benjamin Daniels:** Methodology, analysis, writing, visualization, investigation. **Ada Kwan:** Methodology, conceptualization, analysis, supervision, administration, investigation, funding acquisition. **Vaibhav Saria:** Conceptualization, writing, investigation. **Ranendra Das:** Methodology, analysis, resources, writing, supervision, administration, investigation, funding acquisition. **Madhukar Pai:** Supervision, administration, investigation, funding acquisition. **Jishnu Das:** Conceptualization, methodology, analysis, resources, writing, supervision, administration, investigation, funding acquisition.
