# Supplementary material for: Simulated patients and their reality: An inquiry into theory and method
Source: Soc Sci Med. 2022 May;300:114571. doi: 10.1016/j.socscimed.2021.114571 (PMC9077327; doi:10.1016/j.socscimed.2021.114571)
Supplement: Multimedia component 1 [file mmc1.pdf]

All Narratives Translated from Hindi
Narrative 1
Qualification: AYUSH

Visit 1

On the first visit, when I went to this clinic, I saw that the doctor was sitting on his chair and reading the newspaper. He saw me and asked me to sit. I sat on a stool that was there. He asked, "What is the problem?" I told him my problem. "Doctor Sahib, I have a lot of cough which is not getting better and there is some fever". Then the doctor asked, "Since when do you have the cough?" I answered, "I have a cough since the last two-three weeks". Then he asked, "Do you have fever too?" I said, "Yes". He gave me the thermometer to insert in my mouth. I refused and he said, "Put it in your armpit". I did that. He checked the fever on the thermometer. Then he got up and went to another room which was visible through the glass partition. I could see two beds laid out on one side of the room and in a corner, I could see a cupboard in which the medicines were stored. He assembled the medicines there and came out. He then placed all the medicines on the table and explained that three-three pills each have to be taken in the morning, afternoon and at night. He instructed me to take the syrup along with the pills. He asked me to return the next day. I asked "How much?" He said, "Rs.50". I gave the money and left. While I was there the doctor did not keep any notes or maintain any records on my case. No other patient came while I was at the clinic.

Visit 2

The second day when I went to the clinic, the doctor was assembling medicines in the inner section of the room which was visible through the glass partition. A patient was standing with him. The doctor came out from the inner chamber and standing next to his table, asked – "What is the problem?" The other patient also stood there. I said, 'The medicines you gave me did not lead to any improvement'. He seemed not to have remembered my earlier visit. I was coughing, so he said, "Since when do you have this cough?" I answered, "Since two-three weeks". Hearing this he went to the inner chamber and brought out the medicines. Placing the medicines on the table in three piles of different pills, he explained that I was to take one pill from each three times a day – once, in the morning, once in the afternoon and once at night. He instructed me to take the syrup along with the pills. Then I asked, "How much?" and he again asked for Rs.50 and said, "Come back, tomorrow". I paid and came out and he began talking to the patient who was waiting. Only one other patient had come to the clinic while I was there.

Visit 3

On the third visit when I went to his clinic, the doctor was reading a newspaper. When he saw me, he said, "Come, sit". I said, "Doctor Sahib, the medicines you gave me have not led to any improvement". He asked, "What is the problem?" I told him, "I have a lot of cough that is not getting better." He remarked, "You had come yesterday too". I said, "Doctor Sahib, since last two days I have been taking the medicines you gave, but there is no improvement". He did not say anything but went inside the other room and bought out the medicines. He gave a larger bottle of the cough syrup. He explained that I was to take the medicines three times in the day along with the syrup. I then said, "Doctor Sahib, there is no improvement – Is there anything to worry

about?" He said, "It is so cold these days and in this cold the medicines do not work properly". I asked, "How much"? He asked for Rs.100 today. I paid and left. The doctor did not give me any prescription for medicines to be bought from the chemist. And today there was no other patient in the clinic.

Narrative 2
Qualification: AYUSH

Visit 1
On my first visit to the clinic the doctor was sitting on his chair and looking at his mobile phone. There were no other patients at that time. When the doctor saw me, he said, "Come and sit down". I went and sat on the stool and then he asked, "What is the matter?" I said the opening line, "Doctor Sahib, I have cough since sometime which is not getting better and have some fever too". Then he asked, "Since how many days?" I said, "Since two-three weeks". Then he asked, "Are you producing any phlegm?" I replied, "Yes". He asked, "Do you have cold?" I said, "No, I do not have cold." He then asked, "Do you have body pain?" I said, "No". Then listening to this account, the doctor said, "You have cough since so many days and you have not taken any medicine?" I replied, "I took a cough syrup and some pills from a chemist but did not get any relief". Then he picked up the thermometer from the bottle and asked me to put it in my mouth. When I refused, the doctor put back the thermometer in the bottle. Then he examined my back with the stethoscope and asked me to take deep breaths. After the examination. He wrote a prescription for medicine on a slip and handing it to me said, "Go and get the medicine." I went and got the medicine from a nearby pharmacist which cost Rs.150. The doctor also dispensed a day's medicine from his side and told, "These are medicines for a day, take them three times in the day and also take the medicine you bought from the chemist". He then said, "Report back tomorrow". I asked, "How much money?" He said, "Rs.50". I gave the fee and was about to leave when another patient entered the clinic. The clinic was not very spacious and the doctor's table was next to the door. Some books, stethoscope and a thermometer were lying on the table. Behind a partition a cupboard was visible which was used to store medicines. The doctor spoke very politely to me.

Visit 2
When I reached his clinic, a patient was sitting on the bench. The doctor asked him, "How is your pain?" The patient told, "Pain is less than before but not completely okay!" Then the doctor said, "You take the medicine today and you should feel better soon". The patient then left and it was my turn with the doctor. The doctor asked, "What is the matter?" I said, "Doctor Sahib, the medicine you gave me did not lead to any improvement". Then the doctor said, "I gave you such good medicines, how can you not feel better? It's not possible!" Then the doctor scolded me and said, "For how long will I give you medicines, by changing them again and again? You are saying that your cough is of two or three weeks, I will not give you medicine, get your x-ray and blood test done, only then will I give you any medicine". He further told, "Okay, till the time you get the test reports, I will give you some medicine". Then he assembled the medicines and giving them to me told, "Take these medicines three times a day". After this he

gave me two other pills and said, "Suck these tablets and do not chew them and If your throat feels better with it then buy this tablet from a chemist". Then he wrote out the names of the required tests on a slip and handed it to me. He ever you want". I asked the doctor, "How much money do I have to give?" He said "Forty rupees". I gave the money and came out. The patient, whom the doctor had attended before me, came back and went and sat with the doctor.

Visit 3
When I went to the clinic, I saw there were no patients in the clinic and the doctor was sitting idle. I offered '*namaste*' (greeting with folded hands) to him and sat down. I said, "Doctor Sahib, the investigations you had asked me to get done, I have got them done". Saying this I took out the reports from my bag and handed them to the doctor. First I handed him the blood report, the doctor read it closely and looked at me but did not say anything. Then I gave him the x-ray. He looked at the x-ray for a while and then he said, "Do you have fever?" I said, "It comes and goes". In the middle I was coughing as well. Then the doctor said, "See! I cannot assure you that you will get okay with my medicines. I have read your report and the report says you have TB!" Do one thing, go to a Government Hospital". "Near, ________, there is '________ Government Hospital'; there they will give you treatment. I will not give you any medicines. He then asked me to leave. I asked him, "How much money do I owe you?" He said, "Nothing". I then came out of the clinic. As I was leaving another patient came to him and the doctor started talking to him.

Narrative 3
Qualification: AYUSH

Visit 1
The clinic is located on ________ on the main road and one can read '___________' written on its glass window. The clinic appears small from outside but on entering it is spacious. Two benches are laid for patients on the either side of the entrance to wait their turn. Mounted on the wall above one of the benches is a wooden showcase where small medicine bottles are displayed and it also has cobwebs hanging from it. Further down the room is the doctor's table on which a thermometer, a stethoscope, a torch, an automatic Blood Pressure Monitor and some book s are lying. The table has dust on it. Beyond the table is a sliding door behind which one can see a cupboard from where the compounder assembles the medicines and a bed is also laid out for the doctor to examine the patients.

When I arrived at the clinic two patients were waiting their turn. On my turn, I went and sat on the patient's stool and told, "Doctor Sahib, I have a cough which is not getting better and have fever too". The doctor said, "Since when do you have cough?" I said, "Since two to three weeks". He asked, "Have you taken any medicines for it?" I replied, "Yes, I took some pills and syrup from the chemist". The doctor then gave me the thermometer, which I put under my arm and gave it back to the doctor after about one to two minutes. After this doctor examined my chest with the stethoscope and then asked the compounder to assemble my medicines. The compounder handed them to the doctor after assembling them. The medicines were for one

day, to be taken three times in the day. After this the doctor wrote a prescription for a cough syrup and for some pills and asked me to purchase them from the chemist. I asked the doctor his fee and which he said was fifty rupees. I gave the money and came out of the clinic. No other patient had come to the clinic while I was there.

Visit 2

When I reached the clinic, I saw four patients were waiting before me. When my turn came I said, “Doctor Sahib, the medicine I had taken from you did not result in any improvement”. The doctor asked, “Do you cough all day?” I told him, “I have some cough throughout the day but it gets worse in the morning and at night” The doctor asked, “How is your fever?” I said, “The fever comes and goes in the day but it increases in the night”. Then doctor wrote something on a slip and said, “Do you have the syrup I prescribed for you last time?” I said, “Yes, I have the syrup”. The doctor said, “This is another medicine go and get it from the chemist and till then I will assemble your medicines”. I went and bought the medicines which cost sixty rupees. The compounder passed the assembled medicines to the doctor. They were for a day and the doctor explained me the regimen. When I realized, the medicines were for a single day, I asked the doctor, “Can I get them for three days”. He said, “Yes, you can!” The compounder then assembled three different medicines for three days and explained that I was to take one pill of each kind three times a day and to drink warm water”. I asked the doctor his fee which he said was a hundred rupees”. I gave the fee and came out of the clinic. During this time, no other patient came to the clinic.

Visit 3

When I reached the clinic today after three day gap I saw one patient in the clinic. On my turn the doctor asked me to come and sit on the patient’s stool. I went and sat on the stool and said, “Doctor Sahib, the medicines you gave me did not help”. He asked, “Since when do you have cough?” I said, “Two to three weeks”. The doctor said, “Your cough should have got better by now but since it is not better, we should get an x-ray done to find out why your cough and fever are not getting better”. I said, “Ok, Doctor Sahib, I will get it done, can you write the test?” The doctor did not do any physical examinations. He picked up a pink colored booklet which was of some laboratory and after asking my name, wrote it on the booklet, and said, “Report back tomorrow with the x-ray”. Then the doctor asked the compounder to assemble one day’s medicine for me. The compounder handed my medicines to the doctor and who then told me, “Take this medicine but definitely report back tomorrow with the x-ray, take the medicines three times a day”. I asked the doctor his fee and he said, it was forty rupees. I gave him the fee and came out coughing. One patient came to consult the doctor.

Visit 4

Today when I went to the doctor’s clinic with my x-ray, I saw one patient waiting ahead of me. After he left, the doctor called me and I went and sat on the patient’s chair and gave the X-Ray to the doctor. He took out the X-Ray film from its envelope and after looking at it said, “From where have you got it done?” I said, “I had gone to my brother’s house so I got it done from there itself”. The doctor took out the report and reading it asked, “Have you ever felt this way before?” I asked him, “Felt what, Doctor Sahib?” He explained, “Have you had cough like this

before?" I said, "No". Then the doctor said "Have you ever had TB?" I said, "No". The doctor then advised me, "You should get a consultation from a chest clinic once and they will check your report thoroughly. And if they want to treat you there you should get treatment there. But if they say your report is fine then you can report back to me and get medicines from me". I said "Okay." I told the doctor, "Can you please write down the name of the chest clinic where I have to go for consultation? Otherwise I might forget." The doctor wrote a reference to a chest clinic and gave it to me. The doctor then asked, "What medicines do you need right now?" I told him, "Doctor let me consult the clinic first, and then I will come back and take the medicine from you. He said, "Fine that will be alright. First consult the chest clinic". The doctor didn't take any fee from me. There was no patient after me. I came out of the clinic coughing.

Narrative 4
Qualification: AYUSH

Visit 1
The doctor's clinic was situated in the interior of the main bazaar of the _________. The clinic appeared quite spacious and clean. On entering the clinic one finds two benches laid out on both the left and the right side of the entrance for the patients to sit on. The waiting area has a television mounted on the wall. The compounder's dispensary could also be seen behind and the doctor's cabin with a table on which things like a torch, some books, medicines, a thermometer and a Blood Pressure Monitor were kept. There was also a stool, for the patient. Toward the left-hand side, there was a bed on which a patient could lie down for an examination. There was a patient before me, who came with her mother. When my turn came, the doctor asked me to sit down on the patient's chair and then the doctor asked, "What is the matter?" I said, "Doctor Sahib, I have a cough, which is not getting better and have fever too". Then the Doctor asked me "Where have you come from", I said "From _________". The doctor gave me a Thermometer, which I put under my arm. After checking my fever, he examined my throat and said, "Do you produce lot of mucous?" I said, "Yes". Then he asked, "Have you taken any medicines for this?" I said, "Yes! I took some from a chemist. He gave syrup for cough and some pills for fever." The doctor responded, "You should not take medicines from a chemist as the disease can get worse". Even the previous patient had taken medicines from a chemist which worsened her condition. She came all the way from 'Wazirabad' to get her medicines." After this the doctor wrote some medicines on a slip and asked me to get them from the chemist. I went and bought the medicines from the chemist which cost seventy-five rupees. When I came after buying medicines, I saw that the doctor was standing outside his clinic and smoking a cigarette. He saw me and came inside his cabin while still smoking. The doctor had assembled my medicines from his side too and taking the medicine which I had bought prepared three doses and told me, "Take one dose three times a day." I inquired about his fee. He said, "Eighty rupees", with instructions to report back the next day for next batch of medicines (refills). I gave the fee and came out of the clinic.
I did not like one thing about this doctor. I had told him that I was having a coughing but despite knowing this problem, he continued smoking as he explained my medications to me.

Visit 2
On the second visit to the clinic I saw that the doctor was talking to someone standing outside his clinic. I coughed and went inside the clinic and the doctor followed me. There were no patients waiting but the television was on. I told the doctor my problem with the opening line, "Doctor Sahib, the medicine you gave yesterday, did not lead to any improvement". He told me, "How will it help, there is lot of infection, continue taking the syrup". The doctor stood right there and shined a torch into my throat to examine it. After the examination of the throat, he wrote a medicine on a slip of paper and said, "Today I have written a different medicine, go and get it from the chemist". Then he asked, " Do you still have any of the syrup left from yesterday?" I said, "Yes". I then went and bought the medicines from the chemist. These were two different medicines, four pills each and a bit expensive than what doctor had prescribed yesterday. They cost Rs.108 today. I then went back to the doctor with the medicines and saw the doctor was sitting in his cabin and assembling my medicines. I went and sat on the patient's chair and handed the medicines to the doctor. He said, "These are medicines for one day. Take one dose now, one in the evening and one at night." Take the medicines with warm water and avoid taking a bath tomorrow. Come back tomorrow to get the medicines before one o'clock". I said, "Fine". Then I asked the doctor his fee, and he told me, "Eighty rupees", which I gave and came out of the clinic. Till I left the clinic there was no other patient.

Visit 3
Today when I went to the clinic, I saw that the television was on and the doctor was talking with someone. On seeing me he came inside his cabin and while standing asked, "What is the matter?'. I told the doctor while coughing, "Doctor Sahib, I have not got any relief". The doctor said, "Even after taking such good medicines, you did not feel better! you should get a blood test done". Then the doctor wrote my name, age, a few tests and a medicine on a slip and handing it to me said, "Go and get this syrup from the chemist and I will also give you medicines from my side". I went and bought the syrup from the chemist which cost Rupees ninety-seven. When I took the syrup back to the clinic I saw that the doctor was talking to a patient. After that patient left, I went and gave the syrup to the doctor. He then gave me some medicines from his cabinet and explained that I was to take one dose now, one in the evening and one at night. The doctor then picked up two big pills from his table and said, "This is a table that you suck for reducing irritation to throat. Take these tablets and the syrup, three times a day, one spoon every time if you are having a cough". Handing me the slip, the doctor said, "Get these tests done and report to me tomorrow, you would not be able to get these tests done today as all labs are closed, today being Sunday, so get these reports with you tomorrow". I said "Fine". Then I asked the doctor his fee. He said, "One hundred and five rupees". I gave the fee and came out.

Narrative 5
Qualification: AYUSH

Visit 1
On reaching this clinic, I found that it was very dirty. There was a lot of dirt piled outside the clinic. A cow was standing outside and there was cow dung spread all around the entrance of the clinic. Because of the cold weather the doctor was sitting in his chair with a blanket around his legs. There were some medicines, a stethoscope, a thermometer and a Blood Pressure Monitor on the doctor's table. The doctor was himself assembling the medicines and giving it to patients since there seemed to be no compounder there. I saw that the doctor was attending to an old patient who was accompanied by a younger woman. The doctor assembled the medicines and handed it to the lady and who asked the doctor, "How much is the cost?" The doctor Said, "One hundred and twenty". Then the woman asked if he could reduce the amount? He replied, "This pill costs twenty-seven rupees which I have purchased for myself, that's why I'm giving it to you". The doctor said it would not be possible for him to reduce the fee and then he again asked for hundred and twenty rupees which the lady handed to the doctor. After this, he attended to another patient before my turn.

I went and sat on the patient's chair and the doctor asked. "What is the matter?" I recited the opening line, "Doctor Sahib, I have a cough which is not getting better and I have fever too". The doctor asked me to turn around and checked my back moving the stethoscope up and down, two or three times and asking me to breathe deeply. He asked me if I was taking any medication from somewhere else. I said, "I have taken some medicines from the chemist". He then took the thermometer from his table and asked me to put it in my mouth. I told the doctor, "I feel queasy!" So he put the thermometer under my arm and after a minute took it back to check the temperature. Without saying anything about the fever he said, "You have to take the medicine for five days, do not stop in between." I said, "Okay Doctor Sahib!" Then the doctor asked me "Do you have any difficulty while breathing?" I said, "No". He asked, "How is your fever?" I said "During the day it goes up and down but it increases in the evening". Then the doctor picked up the prescription slip from the table and started writing the medicines. After that the doctor took out some pills from a box on this table started explaining as to how I should take them. He said, "Take one dose in the morning, one in the afternoon and one at night". After this the doctor advised me to avoid eating rice and to drink warm water". The doctor further asked, "Do you drive?" I said, "Yes, I drive a scooter". He advised me to wear a windcheater while riding my scooter. I asked the doctor, "How much money should I pay?" He said, "Rs. One Hundred and twenty". I gave him the money and came out of the clinic.

Visit 2
When I reached the clinic today there were three patients waiting ahead of me but no one came after me. One of the patients was talking with the doctor whom I had seen him at the clinic on my first visit. An old man, who had consulted the doctor yesterday also, was getting an injection administered from the doctor. A lady who had accompanied this person was arguing with the doctor about the injection. She was saying, "Doctor Sahib, yesterday you had mixed the medicine

in the injection from two bottles but today you used only a single bottle". Showing the box of injections to the lady, the doctor said, "See for yourself! Check the cost written on it; this is a more expensive injection than the injection I gave yesterday". He then gestured towards me and asked me to move to the patient's chair. The doctor asked me "What is the problem?" I said, "The medicines you gave me yesterday did not help, there is no improvement". The doctor then picked up the stethoscope from the table and asked me to turn around. He then examined my back with the stethoscope placing it at three-four different places, and said, "You have an allergy". Then, asking my name, he retrieved yesterday's slip from the stack of slips kept in front of him and wrote something on it. Handing the slip to me he said, "This is a cough syrup you need to purchase it from the chemist". The doctor then got up to assemble my medicines from the cupboard kept on the left side of the room. He then arranged the medicines on the table and explained, "Take this one dose now, one dose in the afternoon and one at night". Looking at the medicines I realized that the medicines were for a single day and I said, "Doctor Sahib, coming everyday here will be difficult for me, could I get two days of medicine?" The Doctor without asking any further questions gave me another two days of medicines. The doctor also said, "The syrup has to be taken 10 ml, thrice a day". I asked, "Doctor Sahib, how much money?" He said, "One hundred and forty rupees". I paid and came out of the clinic.

Visit 3

Today I went to the clinic, there was one patient waiting before me and no one after me. The Doctor gestured towards me and asked me to sit down on the patient's chair. The Doctor asked, "What is the matter?" I said, "Doctor Sahib, the medicine you gave, did not help at all, there was no relief I got from it". The doctor picked up a pile of slips and asked me my name, I told him my name. The Doctor then picked up the stethoscope and checked my chest and didn't say anything. After this he asked me, "The syrup I wrote, how of that syrup you had?" I said, "Some of it is still left". The doctor picked up the prescription and asked me if I had any pain in my chest? I said, "No". Now the doctor wrote something on the slip and asked, If I was producing any phlegm? I responded, "Yes, I have it". After this he wrote something on a slip and taking that slip with him, he got up to assemble the medicines. I said again, "Doctor Sahib, there is no relief, I hope there is no cause for worry?" The doctor said, "It is very cold these days, you have an allergic cough". The doctor then assembled my medicine and putting them on the table told me to have one dose now, one in the evening and one at night. I asked, "How much money?" He said, "Fifty rupees". As I was about to leave the clinic, the doctor said, "Report back in the evening". And I said, "Yes, Doctor Sahib".

Narrative 6
Qualification: AYUSH

Visit 1
First day when I went to the clinic, I saw that the doctor was not sitting in his seat but was standing outside his cabin, which is the second section of the clinic. The first section is the patient's waiting area, with two to three benches laid out. The innermost portion had a wooden partition where the medicines were kept in a wooden cupboard. The doctor on seeing me came inside his cabin and told me to sit down. He then asked, "what is the problem?' I said the opening line. "Doctor Saab I have a cough, which is not getting better and have fever too." The doctor did not say anything and gave me a thermometer which he took out of a bottle and asked me to put it in my mouth. I told the doctor, "No", I might vomit". He then took back the thermometer and touched my hand and gauged my fever. Then without saying anything he wrote a slip and asked me to go buy these three capsules from a chemist shop. He did not tell me from which chemist shop. So, I went out and searched for a chemist which I located a lane away. The medicines cost Rupees 18 and I went back and gave them to the doctor. The doctor then arranged the medicines on the table and went inside to assemble the other medicines, which he also arranged on the table and explained, pointing to each kind of medicine, "You have to have one dose now, one in the evening around four o'clock and one dose at night". He then said, "Come after two or three days to report? Then I asked, "Doctor Sahib, can I get medicines for two days?" But he refused and said "No, come back tomorrow and report". Then I asked the doctor, "How much do I have to pay you?" He said "Seventy rupees" I gave the money and came out. The doctor did not keep any records with him. There were no other patients at the clinic when I reached and after I left.

Visit 2
The second day, when I reached the clinic, I saw the clinic was very crowded. Some people were sitting, some were standing and the ones who came after me stood outside the clinic. I went and sat inside on the bench. I went and sat next to the doctor when my turn came. The doctor asked me, "What is the matter?" I said, "Doctor Sahib, the medicines you gave me did not lead to improvement". Then the doctor said, "Yes-Yes, how will you get any relief, it is so cold outside. Do one thing today, take some steam". I said, "I will go home and take steam". Then wrote names of medicine on a slip and said, "Get these three capsules for a day". I said, "Doctor Sahib I will not be able to get off from work every day, can I get medicines for two days?" He instructed the compounder to prepare medicines for three days and asked me to go and get the medicine from the chemist shop. I went and got the capsules for three days which cost fifty-four rupees and gave it to the doctor. The doctor then picked up the medicine his compounder had assembled for me and gave it to me. He asked for two hundred rupees. I gave the money and came out of the clinic. The doctor then attended the next patient. Today there was a larger crowd, that's why he did not say anything; even today he did not record anything.

Visit 3
When I reached the clinic, two patients were waiting before me. At my turn to consult the doctor I went and sat on the stool next to the doctor. He asked, "How are you feeling now?" I said, "Doctor Sahib, the medicine I took from you did not help, there is no improvement". Then he took out a thermometer from a small bottle, wiped it clean and asked me to put it in my mouth. I said, "Doctor Sahib, I will not put it in my mouth." Without a word he put the thermometer back into the bottle. He then wrote the name of the capsule which was the same capsule he had prescribed in my last two visits. He then asked me to go and purchase the medicine from the chemist. I went to buy the medicines and when I returned with the medicine, the doctor was attending to another patient. He asked this patient to go and buy the same medicine. I then handed the medicine to the doctor and who then asked the compounder to assemble medicines for two days for me. The doctor then explained that he was giving you one extra medicine than assembled the medicines. After he completed preparing my medicine I said Doctor Sahib, it has been quite a few days since I have been taking the medicines but there is no improvement; anything to worry about?" The doctor replied, "The other day I had asked you to take steam but you had refused. Had you taken it you would have felt better by now; I have given you very good medicines." I replied, "Doctor Sahib, on reaching home, I had taken hot water steam". On hearing this the doctor remarked, "Oh! That is no steam! Here, at the clinic we would have given the steam with a medicine." He then muttered, "This is what happens, you people do not listen, if you had listened to me then you would have got better by now. I get patients with these symptoms everyday; actually, around 14 patients on an average. You have this problem due to pollution, as there is lot of pollution these days, which we cannot see or perhaps it is because you burst some crackers on Diwali?" He advised, "Whenever you drink water see that it is warm water. Also take your medicine with warm water, you will be fine. There is nothing to worry about - these days we get these kinds of patients". I then inquired about his fee which was Rs.140. He said, "Take the medicines in the same manner as you were taking before." I then paid the fee and came out of the clinic.

Narrative 7
Qualification: AYUSH

Visit 1
The first day when I reached the clinic the doctor sitting on his chair and watching television. On seeing me he asked me to come in and sit down. The doctor then asked, "What is the matter?" I said, "Doctor Sahib, I have cough since many days, which is not getting better and have fever too." The doctor then asked, "Since how many days?" I said, "Since two to three weeks." He said, "You had a cough for so long! Have you taken any treatment?" I said, "I took syrup for cough and some pills for fever from the medical store." He then said sarcastically , "Does a medical store ever treat anyone?" He added, "Get a blood test done, It which will cost two hundred rupees." Saying this he picked up some kind of machine in a box kept on his table. I asked, "Do I need to get the test done now?" The doctor said, "Yes! We will do it now." I told him, "Doctor Sahib, my brother works in a lab, can I get it done from there?" The Doctor replied, "You can get it done from anywhere". The doctor then asked, "Where do you live?" I said, "__________". The doctor

then said, “Get it done from there and you will save some money too.” He then picked up the stethoscope and examined my chest, my back, asking me to take deep breaths. After finishing the examination said, “Your chest seems to be clear”. Then he shined a torch in my throat and examined it. He said, “There is a problem with your throat for which I am prescribing a blood test and a chest x-ray. Report back with the results after two days and do not eat any spicy food”. The doctor then dispensed medicines for two days and assembling three pill doses said, “Have it three times in a day”, adding, “Report back with the test results in two days”. I said, “Ok and asked, “How much money do I have to pay?” He said, “Two hundred rupees”. I gave two hundred rupees and left the clinic. There was no other patient when I left. The Doctor then started working on the computer kept on his table. It was a neat and clean clinic with a separate compounder’s cabin for the lady compounder. Within this clinic, next to the compounder’s cabin was another cabin and on which the name of the clinic, ‘____________’ was written.

Visit 2

When I reached the clinic, the doctor was sitting inside his clinic and a man was standing outside talking on the phone. I went and sat on the patient’s stool. The doctor asked, “You came in the morning too?” I said, “Yes! I had come in the morning but left for my work when I found that you were not available.” The doctor asked, “How are you feeling now?” I said, “The medicines you gave me last time did not lead to any improvement. I had also got the tests done which you had prescribed last time”. I handed the blood report to the doctor and the doctor after reading it said, “You don’t have enough blood in your body”. Then he looked at the x-ray and said, “Your x-ray is showing that you have TB! Does anyone in your family have TB?” I said, “No”. Then he asked, “Does anyone in your neighborhood have TB?” I said, “I have no idea.” The doctor then asked, “Does anyone in your extended family, suffers from TB?” I said “No”. The Doctor then told me, “Do not tell anyone that you have TB. Just conceal it from everyone”. The man who was talking on the phone standing outside the clinic walked in at this moment and went and sat next to the doctor. Doctor then looked at me and said, “First get an investigation done for which the report will be available within seventy-two hours. The investigation will cost two thousand eight hundred rupees. If you get it done from us then we will give you a concession. But it will cost four thousand rupees if you get it done from ‘__________’. And we will start your TB medicine after the results of this investigation”. Then the doctor said, “How many children did you have?” I said, “Two”. “And how old is your youngest child?” I said, “Four years”. The doctor said, “Do not have any more children. A person having TB should not have many children”. He then asked, “What work does your husband do?” I said, “He works in a cloth shop”. The doctor said, “Okay, w’s labhen you report back with the investigations make sure you get your husband along. I will explain him everything”. “A day’s medicine will cost fifty rupees and you can get the treatment from me. It’s not an expensive treatment”. The Doctor told me, “Do not get your tests done from your brother’s lab. Is he your own brother?” I said “No! He is my cousin brother”. The doctor then said, “Then definitely don’t get it done there but from us”. The doctor then wrote a slip and gave it to the compounder saying “Assemble these medicines for two days”. After that the d started drinking tea with his friend. The Doctor’s friend also told me there is nothing to worry about as these days it’s a normal disease. Take your medicines on time. The compounder assembled the medicines for me and explained that these are three packets, and I was to take one packet each three times a day. The doctor said “Today I have added one pill”. I asked, “Doctor Sahib, your

fee?” He replied, “I have increased my fee as the medicine which used to cost fifty rupees now costs three hundred for two days”. I told the Doctor that he had not charged this much money earlier”. He said, “Now I have increased my fee.” I gave him the money and as I was leaving the doctor said, “Come back and bring with your husband with you at least once for sure”. I said “Yes, sure”, and came out. There was no other patient and the doctor started talking to his friend.

Narrative 8
Qualification: AYUSH

Visit: 1
When I reached the clinic, I found some patents were waiting for their turn to see the doctor. There were six patients ahead of me and five patients after me. The clinic was neat and clean and I could see the medicines were stacked in a shelf next to the doctor’s table. There was a glass table top and on it a blood pressure instrument and a thermometer were kept. A lot of visiting cards were kept under the glass. There were two compounders in the clinic. One of them was assembling and explaining the medicine regimen to each patient in turn. The other compounder, who was in the other room, was administering an injection and bandaging a patient. When my turn came, I went and sat on the patient’s chair and the doctor asked, “What is the matter?” I said, “Doctor Sahib, I have a cough which is not getting better and I have fever too.” The doctor asked me to turn around. He examined my back with the stethoscope placing it at three to four different parts of the back, but did not say anything. The doctor then picked up a slip of paper kept on his table and wrote names of some medicines on it. Sliding the paper towards me he gestured toward the compounders to give me the medicine written on the slip. I then asked the doctor, “How much money?” The doctor said, “Fifty Rupees”. I gave him the money. After that I went towards the compounder and handed him the slip. The compounder then assembled my medicines and gave it to me and explained that these medicines were to be taken in the morning, afternoon and evening. I took the medicines and came out of the clinic.

Visit 2
When I reached the clinic, I saw there were eleven patients waiting before me for their turn and nine patients after me. After five minutes of waiting I got a space to sit on the bench, which was kept for patients while they wait their turn. Today I saw that the patients on reaching the clinic were asking the compounder to take out their previous day consultation slip and I also asked the compounder for my slip. He asked me “What time did you come yesterday?” I told him, ”Eleven o’clock in the morning”. After that, the compounder took out the slip and gave it to me. I saw two people relating the symptoms of their child. The doctor inquired about the age of the child and then wrote the name of a medicine on a paper slip and took money from them. When my number (turn) came, I sat on the stool next to the doctor placing the paper with the earlier prescription on his table. The doctor saw my old prescription and asked “Do you have a cough? How is your cough?” I told him “My cough is the same.” I elaborated, “Doctor Sahib, the medicine you had given me didn’t give me any relief at all.” Then the doctor asked me to turn around and put the stethoscope at three different places on my back but didn’t say anything. He wrote the date and the names of medicines on a slip and gave it to me. I asked, “How much

money do I owe you?" The doctor said "Fifty rupees", I gave the money to the doctor and the prescription slip to the compounder; The compounder gave me the medicines in a bag but did not tell me how to take the medicines. When the compounder was assembling the medicines, I saw that these medicines were the same as on my first visit. I took the medicines and came out of the clinic.

Visit 3

When I went to the clinic today, there were thirteen patients ahead of me and ten patients after me. I asked the compounder, to retrieve the slip giving him my name. The compounder asked me my age, I told him, I was thirty-five years old. The compounder asked me on which date and at what time had I come to the clinic? I replied I had come approximately between ten -thirty and eleven in the morning. The compounder searched for the slip inside a bundle of slips and found that slip on the doctor's table. The Doctor asked me what was the matter? I replied that the medicines he had given me did not help. As soon as I said this, the doctor said I needed to get an injection from compounder that he was prescribing. I declined, saying, "Doctor Sahib, I have not had anything to eat, my stomach is empty (*khali pet*)." The doctor deleted the prescribed injection from the slip and made a new slip explaining that I was to take two spoons of the syrup twice a day. The doctor asked me to turn around and he examined me with a stethoscope. He put the stethoscope on my back on three spots, but did not say anything. After that the doctor wrote some numbers and the date of the visit on my slip. At this point I said, "Doctor Sahib there is no relief, I hope there is no cause for worry". The doctor said your cough is because of the cold weather and the pollution. He slid the slip towards me. I asked the doctor how much was the fee. He said it was fifty rupees. I gave him the money and went to the compounder to give him my slip. The compounder assembled my medicines but didn't explain anything to me. Then I took the medicine and came out of the clinic.

Narrative 9
Qualification: Biomedical

Visit 1
The clinic was on the main road and it was quite spacious. When I reached there, the doctor was sitting on his chair and reading the newspaper. A little further away a young girl was sitting there and I discovered, she was the compounder. The clinic was neat and clean. There were no patients ahead of me, so I straightaway went and sat on the patient's chair. Coughing a little I said, "I have a cough which is not getting better and there is some fever too." The doctor asked "Are you taking any medicines?" I told him, "Doctor Sahib, I took a cough syrup from the chemist and he also gave me some pills for the fever." The doctor then asked, "Have you ever consulted me before?" I replied, "No! Doctor Sahib, I have come for the first time to your clinic." The doctor inquired further: "Where have you come from?" I answered, "From __________". He then asked, "Since when do you have the cough?" I told him since two or three weeks. The doctor checked my pulse. He then asked me to unbutton my coat, which I did while coughing. The doctor examined my chest with the stethoscope and then checked my forehead with his hand. The doctor asked the compounder to put a thermometer in my mouth but I refused to do that and told the compounder "I will put it under my arm." The doctor said, "It is okay, put it under your arm." I put the thermometer under my arm. During this time, the doctor started reading the newspaper, sometimes lifting his head and asking questions. "Do you feel a chill when you the fever is rising?" I said "During the day the fever goes up and down but it increases in the evening". Then the Doctor asked, "Do you feel any pain in your arms and legs?" I said, "No". After a few minutes, the doctor asked for the thermometer which I removed and handed it to the doctor. He then asked, "Why did you not consult a doctor earlier?" I replied, "I was tied up with work." Then the doctor asked me my name and he wrote it in an exercise book lying next to him. He continued to question me, "Who has sent you to me?" I told him that my sister-in-law lives close by and that she recommended his name. The doctor asked me her name which I said was "__________". The doctor then asked the compounder to give me an injection and told the compounder what medicines she should assemble for me. When he told her the name of the injection I said, "No, Doctor Sahib, I won't take an injection". He tried to convince me, "If you get an injection, you will feel better soon". I repeated, "No". So, the doctor called the compounder back and told her not to administer the injection but to assemble the medicines. The compounder assembled the medicines and handed them to me. The doctor then said, "This medicine is for one day. Will you come back tomorrow to refill your medicines?" I said "Yes I will come tomorrow". Then the doctor explained how I should take the medicines, "Take one dose now, take another dose in the evening around five o'clock and one dose at night." He told me that he had added another medicine for the fever and when I returned the next day, he would start the medicines for cough. I said, "Okay". Then I asked the doctor his fee and he replied "Seventy rupees." I paid him seventy rupees and came out of the clinic coughing. No other patient came to the Doctor's while I was there. I liked the doctor very much because he was talking very politely to me.

Visit 2
Today when I went to the clinic there was no patient ahead of me. I went inside the clinic and saw that the doctor was reading an Urdu book. I went and sat on the patient's chair. As soon as I sat, the doctor asked me my name and picked up the notebook from his table and opened the page with my name on it. Then he asked, "What is the matter?" I said, "Doctor Sahib, the medicines you gave me yesterday did not lead to any improvement". He said, "Sister, get an injection and you will soon feel better with it". I said, "No" and then the doctor asked me if I had some cough or phlegm. I said, "Yes". He wrote a medicine for cough. I said that the chemist had already given me a cough syrup. The Doctor asked me the name of the cough syrup. I said, "I do not remember the name". He asked, if there was any of it left. I said "No, I finished it all". He wrote the name of a syrup on a slip and asked me to get it from the chemist. Then the doctor wrote something in his copy and gave it to the compounder and told her to assemble the medicines. When the compounder handed me the medicines for one day, I asked the doctor, "Can I get the medicines for two or three days?" The doctor told the compounder to assemble medicines for three days and then explained: "Take your medicines with warm water and in three days you should feel 80% better with these medicines". TI asked about the fee and doctor said, "Two hundred and ten rupees". I handed over the amount and was about to leave when the doctor said, "Come and get your medicines again after three days". I came out of the clinic. There was no other patient waiting.

Visit 3
Today when I reached the clinic there was a patient who had come to ask for his report. The doctor started looking for the report rummaging in the drawer where he kept his papers but could not find it. The compounder too was searching for it. Meanwhile the doctor called me and asked me to sit down. I went and sat on the stool. At this point, the compounder found the report and gave it to the doctor. The doctor took out a small notebook from the drawer and after matching the name on the report with the name of the patient in the notebook, he told the patient "Yes! Your report is here." He gave the lab report to the patient. The doctor asked my name, opened the notebook and asked, "Is the medicine helping?" I said "No". Then the doctor started writing something in the notebook and said, "We should get a blood test done". Then he asked, "Do you produce phlegm when you cough?" I said, "Yes! I do!" He said "Let's do one thing, let's get an X-Ray done first". He brought out a pink pad from the drawer and wrote x-ray on this slip and gave it to me. He then told the compounder to assemble one day's medicine for me. The compounder got the medicines and started explaining the dosage and the schedule for taking the medicines. The doctor gave me the slip on which he had earlier written x-ray, and said, "This lab is only three lanes away from here, when you go to get the x-ray done, tell them to send the report to the clinic. Let me see the report and then I will tell you about the blood test". I said, "Okay". The Doctor said, "Come back tomorrow". I inquired about his fee. He said, "Seventy rupees". I gave the money and came out while coughing. There was no other patient in the clinic.

Visit 4
There was no other patient in the clinic when I came there. The doctor was reading the newspaper. He called me and asked me to sit down on the stool. As I sat I took out the x-ray and handed it to the doctor. After glancing at the x-ray the doctor said, "Why did you not get it done

from the place I had suggested? And how much did it cost?" I said, "There was some problem at my home so I had gone to my mother's house and thus got it done from there itself. And my mother paid the money so I do not know the cost". The doctor looked at the x-ray and read the report. With some annoyance, he said, "Why are you coughing in my face? Turn your face to the other side and cover your mouth with a cloth." He seemed annoyed and said, "The lab I had suggested would have given a bigger X-ray film and on top of that you must have paid more money for this one". He looked at the report again and said, "I told you before, you should get a blood test done. I usually dispense medicines for three to five days and I give injections along with the medicines for three days. But when you did not get any relief from the medicines, I understood that you have a bigger problem!" Next, the doctor asked, "Do you have elevated sugar?" I said, "No". The doctor then said, "Have you ever got your sugar level tested?" I replied, "No". Then the doctor said that you should get a blood test done". I replied, "Doctor Sahib if you could write which tests…". Showing some annoyance again, he said, "Get the tests done here. If these tests can be easily conducted here, then why would I ask you to get them done from anywhere else?" I asked, "How much will the tests cost?" The doctor started scribbling some figures on the newspaper that was kept on his table and after adding the numbers, he said, "The test will cost thirteen hundred rupees, which includes tests for sugar, Typhoid, TB. Eosinophilia and a blood test to find any changes in your blood". I asked, till what time does the clinic operate in the evening. He said, "We do not conduct any tests in the evening, get them done now! I said, "Doctor Sahib, today I am not carrying this much money with me" He said, , "You can pay some amount now and the rest later. What kind of work do you do?" I said that I worked in a beauty parlor. The Doctor then asked me to come around one o'clock in the afternoon to get the tests done. He also said, "You have to get this test done because you have a problem and we will have to start your treatment from today itself. And t you will have to take the medicines for a long time." After saying this, the doctor took out a notebook which had records of my previous visits. After he finished making some notes in, he asked the compounder to assemble the medicines. The compounder assembled my medicines for one day and gave them to me. I asked the doctor about the fee, which was seventy rupees. I handed him a hundred rupee note. The doctor did not give me the change in my hands but flung it on the table. I picked up the change and left the clinic. I did not like this rude behavior. I could not see any other patient when I left.

Narrative 10
Qualification: Biomedical

Visit 1
On the first visit, when I reached the doctor's clinic, it looked very neat and clean. Six or seven patients were waiting to get their medicines from the compounder. The compounder saw me and asked, "Where have you come from?" I said, "Near here, from ___________." There was no other patient before me but subsequently two other patients arrived. The compounder asked me to sit on the patient's chair. The doctor asked me my name and age and wrote it on a slip of paper. Then he asked, "What is the problem?" I said, "Doctor Sahib, I have a cough which is not getting better and I have a little fever too". The doctor asked me to turn around and examined my back three times up and down. The compounder asked me to put a thermometer in my

mouth, but as I was about to put it under my arm, the compounder took the thermometer back from me and kept it back on the doctor's table. The doctor wrote the medicines on a slip and kept the slip on the side of the table. The doctor prescribed a medicine as to be bought from the chemist. The compounder handed me that slip and told me, "There is a chemist next door, go and get the medicine from there". I went and got the medicine from the chemist, which cost thirteen rupees and gave it to the compounder. After this he assembled the medicines from the clinic and explained that the medicines were to be taken three times in the day - in the morning, the afternoon and the evening. The compounder asked the doctor, "How much is the charge?" The doctor said, "seventy rupees". Then I gave the money to the compounder and came out of the clinic.

Visit 2
The second day, when I reached the clinic, I saw four to five patients waiting to get medicines from the compounder. One patient was waiting for a consultation who came before me and another came after me. As soon as I sat on the bench, the compounder asked me my name. I said, "_______". The compounder then took out the medicine slip with my name and asked, "How is your fever?" I replied, "It is low". The compounder then placed that slip on the doctor's table. The doctor then called out my name, "______" and t I went and sat on the chair in front of the doctor. He asked me about my cough. I said, "Doctor Sahib, the medicine you gave me did not lead to any improvement". The doctor had his stethoscope around his neck with which he then examined my back placing it at four different spots. After finishing this examination, he wrote names of some medicines on the slip that had my name on it. He informed me that I would need to take medicines for four or five days. This time the doctor prescribed medicines for two days. These were same medicines that I received at my last visit. Today also the compounder asked me to go and purchase the medicines from the chemist, which I went and bought from the chemist next door and handed them to the compounder. The compounder explained that the medicines had to be taken in the morning, evening and night. Then the compounder asked the doctor, "How much money?" The Doctor told, "Hundred and Ten rupees". I gave the money to the doctor and came out of the clinic.

Visit 3
When I reached the clinic, I first asked the compounder to retrieve the slip on which the medicines from the last visit were written. The compounder retrieved the slip and kept it on the table of the Doctor. After seeing the slip, the doctor called for me. When I sat on the patient's chair, the doctor asked, "What is the problem?" I replied, "Doctor Sahib I don't feel better". The doctor asked, "Do you feel a chill (cold)?" I answered, "No". He then asked me to turn my back towards him, and he placed the stethoscope in four different parts of the back (upper and lower back). Then he began to write the medicines on my old slip and put it on one side. I asked, "Doctor I don't feel better. Is there anything to worry about? The doctor answered, "Continue your medicines. The compounder took the slip from the table to assemble the medicines. When the medicines were ready, he called out my name and I went to collect the medicines. The compounder took out the medicines from the envelope and laying them on the table he explained how to take them (the timings and dosage). But the compounder did not tell me that the medicines were for two days. On counting the pills I decided that these were for two days.

Then, looking at what the doctor had written on the slip, he said, come and get steam. I said I have to go to work now. I will take the steam at home. The compounder said “Okay, also drink hot water”. Then the compounder asked the doctor, “How much is the charge?” The doctor said, Rs.140. Then I gave the money to the compounder and left.

Narrative 11
Qualification: Biomedical

Visit 1
On the first visit, when I reached the doctor’s clinic, there was no other patient except the compounder. I went and sat on the patient’s chair. The doctor asked, “What is the problem?” I told him, “Doctor Sahib, I have cough which is not getting better and have some fever also”. Then the doctor asked, “Since when do you have the fever?” I said, “Since two–three weeks”. After this the doctor picked up the thermometer and brought it near my mouth, which I took from him and put under my arm. During this time the doctor asked, “How are your stools?” I said, “My stools are fine”. The doctor then told me to take out the thermometer from under my arm and hand it over to him. The doctor then asked, “Do you sleep well?” I answered, “I sleep well’. Immediately after this the doctor asked, “Do you produce any phlegm?” I said, “Yes”. The doctor then picked up the blood pressure instrument lying on his table and measured my blood pressure with his left hand and put the stethoscope hanging around his neck on my wrist. After measuring my blood pressure, he examined me with the stethoscope by placing it on my chest (front) and at four different places on my back. The doctor then asked, “Where have you come from?” I said, “I live only two lanes from here”. The doctor said, “At whose place?” I said, “At __________’s as I suddenly remembered that one of my friend’s brother lived in the same area. The doctor then asked, “With him?” I said, “I rent it from him”. After this the doctor wrote my name, age and medicines on a plain paper and then handed the paper to the compounder. The compounder then assembled the medicines in a packet and handed them to the doctor. The doctor then took out the medicines from the packet and explained that three pills had to be taken from it in the morning from the envelope marked as ‘Morning’; four pills from the one marked as ‘Afternoon’ and three pills from the one marked ‘Night’. The syrup, five ml each, should be taken in the morning, evening and night. Then, I asked, “How much?” He said, “Rs.300”. I gave the money. After taking the money, the doctor said, “The medicine is for two days and if you have any problem come back tomorrow”

Visit 2
When I went to the clinic today only the compounder was there. I asked him, “Has Doctor Sahib, arrived?” He said, come and sit down Doctor Sahib is just performing his prayers and will finish soon”. After about two minutes the doctor came out from the compounder’s cabin and asked me to come and sit on the patient’s chair. I got up from the bench and went to the patient’s chair. The doctor asked me, “What is the matter with you?” I told, “Doctor Sahib, the medicines you gave me, did not lead to any improvement”. The doctor then asked, “How is your cough?” I said, “Doctor, there is no relief.” The doctor said, “Earlier you must be coughing four times in an hour, and now it must be three times?” I said, “ No doctor Sahib, it is the same.” He picked up the

stethoscope from the table and examined my chest, twice on the left side and twice on the right. While doing this he asked me to take a deep breath. After this he kept the stethoscope back on the table but did not say anything. Then he asked me my name and after retrieving my slip he said, "I am changing one medicine". After this the doctor gave the medicine slip to the compounder. The compounder assembled the medicine in a small paper packet and took it to the doctor. The doctor then took out the medicines from the packet on his table and explained, "Take these medicines in the morning and this one pill, (pointing to it), half an hour later. And repeat the same dose in the afternoon and in the night". And while handing three packets to me, he asked "Are you taking the syrup I had given you earlier?" I said, "Yes." The doctor then said, "If you have any problems then report back to me tomorrow". The medicines were for two days. HE advised me not to consume any fried food. I asked the doctor, "How much do I owe you?" He said, "Two hundred and forty rupees". I gave the money and came out of the clinic.

Visit 3
When I went to the clinic there were two patients waiting before me and one came after me. On my turn, I went and sat on the patient's chair. The compounder was not to be seen today as I saw the doctor himself assembling the medicines for the previous patient. The doctor asked me, "What is the matter?" I told him, "Doctor Sahib, "The medicine you gave me did not lead to any improvement". The doctor then picked up the thermometer and gave it to me. I placed it under my arm. After about thirty seconds, the doctor took back the thermometer. Reading the temperature, he said, "You do not have fever". After this he examined my chest (front) and my back with the stethoscope asking me take deep breaths. Then the doctor asked, "How is your fever? I said, "It varies during the day but at night it increases". Finally, the doctor asked my name and retrieved my slip from the bundle of slips. He then wrote the date of consultation on it and remarked, "Take these medicines and then we will see." I asked, "Doctor Sahib, I have had no relief, I hope there is nothing to worry about?" The Doctor said, "It is an infection, take the medicines". Then I asked the doctor, "How much money I owe you?" The Doctor said, "Three hundred and forty rupees". I gave the money to the doctor and left.

Narrative 12
Qualification: Biomedical

Visit 1
When I reached the clinic, the doctor was attending to a patient and there were two patients who came in after me. The clinic had a cabin for the compounder in the front and then was the doctor's cabin. The clinic was very neat and clean and I saw the medicines were neatly stacked in one of the showcases. When my turn came, the doctor gestured towards me to sit on the patient's chair. The doctor asked me, "Where have you come from?" I said "I have come from nearby". He asked me "From where?" I said, "From ___________." The doctor asked, "Why did not you consult someone there?' I said, "My elder brother suggested I should consult you so I came here." The doctor then asked if I had taken any medicines till now? I said, "I had taken medicines from the chemist". He then picked up the thermometer and asked me to put it in my mouth. As I was about to put it under my arm the Doctor stopped me and said, "Put it in your

mouth". I said, "Doctor Sahib, I will put it under my arm". He then told me, 'There is a wash-basin, go and wash it there". I went and washed the thermometer and came back with it. The doctor then took the thermometer from my hand and asked me to open my mouth and put the thermometer in my mouth. He then removed it after thirty seconds and read the temperature but did not say anything. Then the doctor asked me, "Since when do you have the cough?" I said, "Doctor Sahib, it has been two to three weeks". After this he picked up an ice cream stick kept on his table, and while pressing my tongue with it, he examined my throat with a torch. Then he picked up the stethoscope from the table and asked me to turn around. He then asked me to lift up my shirt and then examined my back once and said, "There doesn't seem to be anything". After this the doctor took a printed pad, asked me my name and age and wrote something. He said, "I am prescribing medicines for five days, if you do not feel better then I will prescribe a blood test and an x-ray . The doctor then explained the dosage of the medicines he had written on the slip and said, "Have this medicine thrice a day, this second one, once a day and the third one, three times a day. Take the syrup, 5ml, with hot water; along with this take steam twice a day". I asked him "How much money do I have to give you?" The Doctor said, "Three hundred and fifty rupees". I gave the money to the doctor and came out. He did not dispense any medicines which was different from other doctors

Visit 2

When I reached the clinic, the doctor was attending to a patient. There were two other patients waiting before me and another three came after. When my turn came, I went and sat in the patient's chair. The doctor asked me, "What is the matter?" I showed him the previous prescription and said, "Doctor Sahib, the medicines you gave me did not lead to any improvement". The doctor again asked, "What is the matter?" I said, "Doctor Sahib, I'm having a cough and fever". The doctor picked up the thermometer from the table and put it in my mouth for and took it out after about forty seconds and said, "There is no fever". Then he asked, "How is the fever?" I said, "Doctor Sahib, fever goes up and down during the day but increases in the night". The Doctor then asked me to open my mouth and put the ice-cream stick on my tongue and examined my throat by shining a torch. He then said, "There is an infection; I am writing a medicine for five days, it should give you relief!" Then the doctor picked up the stethoscope from the table and asked me to turn around and also asked me to lift up my shirt and examined my bare back with the stethoscope. The Doctor then asked me, "How is your urine?" I said, "It is fine". Then the doctor asked, "What type is the phlegm from your nose? Is it yellow or white?" I said, "White". After this the doctor wrote a prescription on a slip and explained that he was changing some medicines. He said, "These antibiotics that you have taken, take them for another two days as we do not give antibiotics for more than seven days". The Doctor then wrote medicines for another two days and also explained that I should take these in the morning and evening. He said, "Take the syrup, five ML morning, noon, evening." After this the doctor wrote a prescription for a few tests and said, "Although I don't see anything wrong, if you don't get relief then get these tests done and report to me after five days". After this I asked the Doctor, "How much should I pay you?" He said, "Three hundred and fifty rupees". After paying the money I came out of the clinic.

Visit 3
When I reached the clinic, there was one patient s waiting before me and one came after. On my turn, I went and sat on the chair opposite the doctor. I took out the x-ray and the CBC reports from my bag and handed them to the doctor. The doctor asked, "Have you got your previous prescription?" I then handed the old prescription slip to the doctor. He looked at the CBC report and said, "You have TB!" The Doctor then asked, "Where have you come from?" I said, "Doctor Sahib, from __________." The doctor said, "But this report is from ____________?" I told, "Doctor Sahib my brother works there. So I had the test done there". The doctor looked at my report and asked me if I understood English. I said, "No". After this he called the compounder and asked him to get an injection and turned towards me and said, "I will now administer an injection." I told the Doctor," But, I have not eaten anything." The Doctor said, "No problem! This injection is administered on an empty stomach". I said, "Doctor Sahib, can you prescribe a medicine for now and I will come back later for the injection." After that the doctor picked up the thermometer lying on his table and put it in my mouth and removed it after thirty seconds and returned it to his table. He then held the x-ray against the tube light and said, "According to the x-ray you have TB.". The doctor then said, "We will get your sputum test done as we do not know clearly from an x-ray if it is TB." After that the Doctor took out the Ice-cream stick and the torch from his table drawer and examined my throat by putting the ice-cream stick on my tongue and shining the torch into my throat. The Doctor asked me "Who all are at your home?" I said, "My brother, sister-in-law and my mother". The doctor asked me, "Does anyone have cough in the house?" I said, "No". Then the Doctor asked, "Are you producing any sputum?" I said, "Yes, Doctor Sahib." Then the Doctor said, "Is there any blood in the sputum?" I said, "No, Doctor Sahib." After that the doctor asked the compounder to get the Monteux test kit to his table. The compounder went and got the kit and put it on the table. After that the doctor said, "I will do this test, it's called a Monteux test and gestured me to put my arm on the table. I said "Doctor have not eaten anything can you prescribe the test and I will get it done from another lab". Then the doctor picked up the stethoscope from the table and asked me to turn around. He asked me to raise my shirt and then examined my back with the stethoscope. He said, "For now I am writing for a sputum test for which you will have to come here for three days to give the sputum sample. After that I will get a Monteux test done." After this doctor wrote some medicine on the prescription pad and pointing to the names he had written said, "Take this medicine right now. I am giving you medicines for five days. Have the first medicine once a day and the second one three times a day. The last one is a syrup, take it three times a day with warm water." After that the doctor told me, "This is an iron test, get this one done too". I said, "Okay, Doctor Sahib". After that I took out five hundred rupees from my pocket and gave it to the doctor and the doctor deducted three hundred and fifty rupees and returned one hundred and fifty. I came out of the clinic.

Narrative 13
Qualification: Biomedical

Visit 1
On my first visit to the clinic I found one patient ahead of me who was waiting for his medicines. The doctor was assembling the medicines as they conversed. The doctor invited me in. I came in and sat on a bench. The clinic is a small room with a table and chair at the e front a blue plastic curtain that divided the room Behind the curtain was a bench and a small wooden cupboard with medicines. After handing over the previous patient's medicines, the doctor came and sat on his chair. He asked me, "What is the matter?" I told him, "Doctor Sahib, I have a cough which is not getting better and I have fever too". The doctor picked up a thermometer from a glass bottle and dipped in a water bowl placed on the table. After flicking off the excess water he asked me to put the thermometer in my mouth. I refused; so, he said, "Okay, placet it under your arm, so that it touches your skin". Then he placed the stethoscope on my back for a minute. Taking the thermometer back from me, he said, "You don't have fever at the moment." I told him, "The fever comes and goes". Then he wrote a prescription for medicines on a prescription slip and said, "Here is the prescription for medicines for three days. Buy these from a chemist". Unlike the case of some other doctors, he did not ask me to bring the medicines back to the clinic for confirmation. Then the doctor went to the cupboard, which was visible from where I was sitting. After assembling other medicines, he came back and said, "Take these medicines three times in a day". He then said, "Come back after three days", and "Gargle with hot water". Then he went and sat on his chair. A patient walked in at that time and he asked him to sit down. Then I asked, "How much money?" He said, "Three hundred rupees". The doctor did not keep any records of the case. Because he had a fracture in his right hand and was wearing a support band from neck to hand. I gave him the money and left.

Visit 2
When I reached the clinic, I saw that the doctor was standing outside his clinic, leaning against a car, in the sun, talking to a person. When he saw me, he asked me to come inside the clinic. After I sat on the stool, he asked, "What is the matter?" I said, "Doctor Sahib, the medicine you gave me yesterday did not lead to any improvement". I was coughing, so the doctor said, "You will get relief slowly and steadily but the symptoms will not get better quickly." He immediately asked, "Do you have fever?" I said, "Yes, I have low fever". The doctor then picked up the thermometer kept in a bottle and washed it with water which was kept in a bowl on his table and asked me to put it in my mouth. I took the thermometer and immediately put it under my arm. The doctor did not make any remark, and after sometime asked for the thermometer back, looked at it and put it back in the bottle. He then examined my chest with the stethoscope but did not say anything. The doctor then got up went to the cupboard of the medicines and after assembling my medicines, put four pills on his palm and gave me instructions, "Take these four medicines three times in the day". I noticed he had added a new pill to the ones he dispensed yesterday. The doctor then sat on his chair and wrote the name of a medicine on a slip of a paper and said, "Purchase these six capsules from the chemist and take one capsule each in the morning and at night, and when you come back after three days bring this slip with you". I had forgotten to bring the slip from my first visit which is why he gave this instruction. Then I asked, "Doctor Sahib, how

much is your fee?” He said, “Rs.300”. I paid and left the clinic. There were no other patients at the clinic today.

Visit 3

When I reached the clinic, I saw the doctor talking with a man, who left when he saw me and went to a house next door. The doctor asked me to sit and said, “What is the matter?” I told him, “Doctor Sahib, the medicines you gave me did not lead to any improvement and it has been quite a few days since I am taking the medicines.” He said, “Don’t worry, you will get some relief as you continue to take the medicines and it takes time to getter better”. I was coughing, I handed him the prescription slip for the medicines from my previous visit. He asked, “Do you produce any phlegm?” I replied, “Yes, I produce phlegm”. He then asked, “What colour is the phlegm?” I said, “White”. The doctor then remarked, “You have an allergy, continue with the medicines”. He then got up to assemble the medicines from his cupboard and I again said, “Doctor Sahib, I did not get any relief, hope there is nothing to worry about?” He then came near me and while putting a dose of medicine on his palm said, “Take these medicines three times a day”. Then looked at me and said, “Looking at you it does not appear that you have TB but for your satisfaction you should get an x-ray done. There is _________ in _________, get the x-ray done from that lab”. I asked, “Doctor Sahib, my brother works in a lab, can I get the x-ray done from that lab?” He replied, “Okay, get it done from there but tell them to do it properly and when you get the result, report it to me”. I then asked the doctor,” How much money do I have to pay?” He said, “Rs.300”. I paid and came out of the clinic. There was no other patient in the clinic today.

Visit 4

When I reached the clinic, I saw a lady was sitting in the clinic. The doctor saw me and said, “Sit, I will attend to you soon”. Then he went and sat on his chair which was a little further away and I went and sat with a woman who was sitting on the bench. The woman asked me, “Have you come here to get your medicines? I said, “Yes” while coughing. She murmured softly, “Go to the Government Hospital where you will get free medicines and quick relief! This doctor is no good as the medicines he dispenses give no relief and he is expensive too! I then asked the woman “Are you here to get some other task done?” She said, ‘Yes, I have come to get a medical certificate”. The doctor came with four medical certificates for which she paid Rs.840 and then she left the clinic. The doctor called me to come and sit on the patient’s chair and asked, “What is the matter?” I said, “Doctor Sahib, you had asked me to get an x-ray done, which I did.” I took out the slip from the previous visit and put it on his table. He read the slip and said “Yes, Yes!” I took out the x-ray film and the report from the bag and handed them to the doctor. After looking at the x-ray film he said, “Your x-ray is fine”. Then he read the report and said, “You have TB”. After this he read the blood report and said, “Your ESR is fifty! It should have been fifteen”. The doctor asked, “Do you produce any phlegm?” I said, “Yes! I do.” “What color is the phlegm?” I replied, “White”. The doctor paused and said, “I am prescribing a sputum test”. And then, pointing at the slip, he said, “You did not get this test done, the test where they put a mark on the arm?” He showed me the spot on his arm where the test was to be done. I said, “This test? I was told to come and get it done today. I will and get it done”. The doctor said “Fine, get it done today and also get the sputum tested for which the results will be available in two days.” Then he wrote a medicine on the slip and pointing to it told me, “See this medicine has four pills in a

strip, it is a TB medicine which you can buy from the medical store. You have to take all the four pills together on an empty stomach in the morning.” Then he said, “Don’t start it yet. Wait for the test results.” Then he said, “Okay I will give you medicines for two days”. I said, “Doctor Sahib, I still have one day’s medicine left.” The doctor then told me, “Okay, we will start the treatment after your test results.” I then asked, “From where will I get the medicines?” The Doctor said, “From my clinic and you will have to take the treatment for 6 to 8 months.” I asked, “Doctor Sahib, are the medicines expensive?” He replied, “The treatment for TB is not expensive. It’s cheap!” The doctor said, “First get the tests done and report back with the results.” He did not charge any fee today. Another patient entered the clinic as I left.

Narrative 14
Qualification: Biomedical

Visit 1
Today when I reached the clinic and entered through the main door, I saw that the clinic was part of a nursing home. There was a reception area on one side, a dispensary on the other and four to five other rooms. One of these rooms was a Lab, another room was for x-rays. Other rooms were for inpatients. One lady was being administered an IV drip in one of the rooms. Some people were waiting at the reception area. The doctor’s cabin was on the right hand. On entering the cabin, I saw that it was not clean, with medicines, injections, thermometer, pens, B.P. Monitor and notepads scattered on the doctor’s table. A male and a female doctor were sharing the cabin for consultation. I saw two female patients in consultation with the female doctor. A showcase with boxes filled with branded medicine strips was visible. The boxes had labels, such as B.P, Sugar, Allergy, Ladies, written on them. Both the providers were dispensing medicines from these boxes. Cotton stubs, medicine wrappers, injection wrappers, were seen strewn on the floor. A compounder was also present in the cabin who took my name, age and the problem on a piece of paper. Then the compounder gave me thermometer which I put under my arm. He asked for it after a while and wrote the temperature on that paper and handed me the slip. Two patients were waiting for their turn before me. On my turn, I went and sat on the patient’s stool and gave the slip to the doctor. I said, “Doctor Sahib, I have cough since one or two weeks which is not getting better and I have some fever too”. Then the doctor asked, “Since when do you have the cough?” I said, “For the past two or three weeks.” Doctor asked, “Have you taken any medicines for this?” I said, “Yes”. The doctor’s phone rang and after attending the call he again asked me “Since when do you have fever?” I said, “Since two to three weeks”. Then the Doctor examined my chest and back with the stethoscope. After this, he examined my throat by shining a torch and asked, “Do you get headaches?” I replied, “No”. He measured my blood pressure and wrote something on the prescription slip. Then he asked, “Have you taken any medicines?” I said, “Yes, Doctor Sahib I had taken medicines from a chemist who had given a syrup for my cough and some pills for fever.”. The doctor started writing something on the prescription slip and asked, “Where have you come from, I hope not from far away?” I told him, “From A block”. Then the doctor gave me a syrup and asked, “Should I dispense medicines for one or two days?” I said, “For two days”. The doctor then wrote the name of the medicine on the prescription slip and said “Get these medicines from the compounder”. I asked the doctor his fee. He read the cost of the syrup and

replied, “Two hundred and fifty rupees”. I gave the fee and came out of the cabin coughing. Five patients were waiting when I came out of the cabin. I then went and handed the prescription to the compounder in the dispensary. The compounder assembled the medicines and explained that the medicines were to be taken three times a day. He asked me if I had paid the fee? I said “Yes, I have paid it”. Then I came out of the clinic.

Visit 2
Today when I went to the clinic I saw five patients waiting before me. I went and sat inside the doctor’s cabin. The compounder retrieved the slip from the previous visit after asking my name and gave me the thermometer which I put under my arm. The compounder asked for it after a minute. After reading my temperature he wrote it on the slip and handed me the slip. I took the slip and waited for my turn. On my turn, I went and sat on the patient’s chair and said, “Doctor Sahib, the medicine you gave me did not lead to any improvement”. The doctor asked, “Is there any relief in cough?” I said, “No”. Then the doctor said, “When do you have more cough?” I said, “Off and one through the day but more in the morning and night”. The doctor told me to take steam and started to explain how to do it. He again asked, “Have you had fever?” I said, “Yes”. He asked “Any pain?” I said, “No”. Then the doctor checked my pulse and examined my chest with the stethoscope. Then he wrote some medicine on his slip and said, “I will administer an injection today” but I refused to take an injection. The doctor then wrote the names of some medicines on the slip and asked me to give it at the dispensary outside. I asked the doctor his fee. He said, “Two hundred rupees.” I paid the fee and came out of the cabin. I gave the slip to the compounder and after assembling my medicines, he asked me to show the medicines to the doctor for confirmation. I went back to the doctor’s cabin. He looked at it and said “It’s okay! Take them.” There were five patients who were waiting when I left. I took my medicine and came out of the clinic.

Visit 3
When I reached the clinic today there were six patients waiting ahead of me. I went and sat with them. A female compounder was already there and she asked me my name and retrieved my previous slip after glancing through it. She then gave me the thermometer, which I put under my arm. After some time, she asked for the thermometer and I gave it back to her. She read the temperature on the thermometer and wrote it down on the slip and gave the slip to me. I held the slip in my hand and waited for my turn. When my turn came, I went in and sat on the patient’s stool. Then the doctor asked, “Tell me what is the matter?” I said, “Doctor, the medicines you gave did not lead to any improvement.”. The doctor then examined my chest and my back with the stethoscope. He then asked if I was taking steam. I said, “Yes.” He then wrote a name of some medicines on the slip and was about to hand the slip to me when I asked, “Doctor Sahib, I hope there is nothing to worry about?” The doctor said, “You should have felt better with the medicine but we can get an X-Ray done”. I said “Ok, if you write it down for me I will get it done”. He said, “We have the facility in the clinic itself so you can get it now.” I told the doctor, “I will come back and get it done in the evening”. Then the Doctor said “Okay”. But he did not give me the slip with the CXR written on it. I then asked the doctor, “Can you please tell that how much it will cost?” He said, “Two hundred and fifty rupees”. The doctor then wrote the names of the medicines and asked me, “Should I give you medicines for two days?” I said, “Yes”. I asked the

doctor his fee which he said was one hundred and sixty rupees. I gave the fee and came out of the cabin. There were four patients waiting their turn. When I came out the compounder was not in the dispensary. The female compounder came after ten to fifteen minutes to assemble my medicines. She gave me my medicines and explained how to take them and that the medicines were for two days. I said “Fine”. The compounder asked me If I had paid the fee. I said “Yes”. Then I came out of the clinic.

Visit 4

Today I went to the clinic with my x-ray. I went inside the doctor’s cabin and today also the compounder asked me my name and retrieved my previous slip ad gave it to me. Two patients were waiting before me. When my turn came, I went and sat on the patient’s chair. The doctor looked at my slip and said, “Do you feel any relief?” I said, “No.” Then he said, “Do you have any relief from fever?” I said, “No”. Then I handed the x-ray to the doctor. He examined the x-ray film and then read its report and said, “Your report says you have Pneumonia”. Then he checked my pulse and examined my chest and back with the stethoscope and started writing something on the slip. He said, “You need to get a blood test done from here, now”. I asked the doctor, “How much will it cost?” He said, “Two hundred and fifty rupees”. Then I said, “Can I come back in an hour and get it done?” The Doctor said, “Ok, you can come back”. Then he started writing my treatment on the slip and said, “Do I give you medicines for one day or two days?” I said, “Doctor please give it for one day”. The doctor then wrote the prescription for medicines for one day and gave the slip to de saying, “When you take this medicine your urine might turn red, but don’t get scared.“ I said, “Okay”. He gave me the slip. I asked the doctor his fee. He said, “One hundred rupees”. I gave the money to the doctor and he then told me, “You should get the blood test done quickly as your x-ray is showing some problem in it and for which you will have to take medicines regularly for a long time”. I said, “Okay”. I came out of the cabin and gave the medicine slip to the compounder. The compounder assembled my medicines and asked that I take them to the doctor and show them to him. I went back to doctor’s cabin with the medicines. The other compounder who was present there took out one packet of the assembled medicines and showed it to the Doctor. The doctor then took out three brown colored pills from his drawer and asked the compounder to add those with the other medicines and also asked to add another medicine from the dispensary. The compounder came out with me and added three white pills. When I left , there were three more patients waiting for their turn.

Narrative 15
Qualification: Biomedical

Visit 1

This clinic was on a wide road. As one enters four long benches are laid for the patients to sit. I went and sat there. The doctor’s cabin was little inside, where the doctor was attending to a patient. In the dispensary two female compounders were assembling medicines for patients. The clinic had glass windows and doors through which main road was visible. Two patients were waiting before me for consultation. When my turn came, I went inside the doctor’s cabin and sat on the stool. After coughing a little I said, “Doctor Sahib, I have a cough which is not getting better

and I have some fever too." The doctor asked, "How long have you had this?" I answered, "Since two or three weeks." The doctor's phone rang and he answered the call. I felt that the doctor had perhaps not heard my answer. After attending the call, he again asked, "For how long have you had the cough?" I repeated, "I have a cough for two or three weeks." He then examined my chest with the stethoscope. Then cleaning and shaking the thermometer, he gave it to me. I refused and said, "Doctor Sahib, I have a cough!" He said, "In in an emergency one can place t it under the arm. So now place it under your arm." I took the thermometer and put it under my arm. I saw that some paper slips for other patients were lying on the table along with some blank slips. The doctor wrote my name on the one of the blank slips and wrote the names of medicines for me. Then the doctor asked me to remove the thermometer from under my arm. I gave it to the doctor. The doctor said, "Come tomorrow for the medicine refills. Right now, I'm giving you medicines for a day. He then opened the drawer of his table and took out a medicine strip and started cutting out two pills from it. Giving the pills to me he instructed, "Give the medicine slip to the compounder and do not eat or drink any cold thing, Avoid rice, pickles and fried stuff. I got up from the patient's chair and took the slip to the compounder. The compounder gave me the medicine and also explained how to take the medicines. I went back to the Doctor and asked him his fee. The doctor said, "Seventy rupees." I was giving him the money when he again reminded me. "Come tomorrow to get the medicines." I said, "Okay." I left the clinic while coughing.

Visit 2

When I reached the clinic, a patient was waiting before me and who had come get his refill for medicine to increase weight. On my turn the Doctor asked my name and retrieved my previous day's slip. I said, "Doctor, the medicine you gave me did not lead to any improvement." Then Doctor asked, "How is the cough in the morning or do you have it the whole day?" I said, the cough is all the time but it is more severe in the mornings and at night." Then the Doctor examined my throat by shining a torch and asked, "Do you have headache?" I said, "No". Then the Doctor asked me if I had pain in my throat? I said "No!" Then the Doctor wrote the name of the medicine on his slip and asked, "For how long do you want the medicine, two days or three days?" I said, "For three days". And while the doctor was writing the medicines the doctor asked me if my house was well ventilated or not? I said, "It is." Then the doctor said, "Then do one thing, when you get up in the morning open all doors and windows so the air inside your house changes and when you sweep the house make sure the doors are open so the stale air does not remain in your house." The doctor took out some strips of medicines and cut some out and gave them to me saying, "Do you use a nebulizer?" I said "No". Then Doctor said, "Take the medicine from the compounder." I took my slip and took the medicine from the compounder and I went and asked the doctor how much his fee; he said two hundred and ten rupees. After paying the fee I came out. I saw there were three more patients in the waiting area.

Visit 3

Today when I reached the clinic I saw that there were two patients waiting before me. When my turn came, I went and sat on the patient's chair. The doctor asked me my name and retrieved my previous slip and said, "What is the matter with you?" I told, "Doctor Sahib, there is no improvement with the medicines you gave." He asked, "When did you take the medicine?" I said, "Two to three days ago." At first the Doctor said, "How will you get relief? You have taken the

medicine from me three days ago. (Here, I felt as if the Doctor felt that I had taken only one day's medicine three days ago) So, I told the doctor, "I had taken three days of medicines from you." Then he remembered. After that he examined my chest with the stethoscope and my throat by shining a Torch. He asked, "Any pain or soreness in the throat?" I said, 'No'. Then the doctor wrote a medicine on a slip and was about to take out some pills from his table drawer when I said, "I hope there is nothing to worry about." The doctor said, "There is nothing to worry about, just do not eat pickles, fried food and take your medicines with hot water. Then he directed me to get the medicines from the compounder. I gave the prescription slip to the compounder and got the medicines for two days in addition to two small bottles of pink colored syrup. Then I went to the doctor and I asked him what was the fee. He said it was one hundred and forty rupees. I gave the fee and came out. I found three more patients in the waiting area.

Narrative 16
Qualification: Biomedical

Visit 1
I went to the clinic and sat in the waiting area. When my turn came, I walked to the patient's chair and sat down. I said, "Doctor Sahib I have a lot of cough which is not getting better, and some fever too". The doctor said, "Do you have any pain in the throat?" I said, "No". Then the doctor asked my name and address and put it on a slip of paper. He asked me, "Since when have you had the cough?" I said, "Since 2 or 3 weeks". The compounder was handing me the thermometer to put in my mouth. I said, "No, I am coughing a lot", to which the compounder responded, "Doesn't matter. Put it under your arm." I put the thermometer under my arm. The doctor started talking to another patient. After 2-3 minutes the compounder took back the thermometer and noted the temperature. Then the doctor examined me with the stethoscope placing it on my chest. Next, he shined a torch in my mouth to examine my throat. Then he wrote the names of medicines on the slip on which my name and address were written and gave it to the compounder. He gestured to another patient to come forward so I got up from the patient's chair and waited for my medicines. After 2 or 3 minutes the compounder put all the medicines on his palm and explained that each medicine had to be taken 3 times a day. He gave me the medicines and asked for Rs.50, which I paid. The compounder said, "You will need to take the medicines for 2 or 3 days".

Visit 2
The second day when I reached the clinic, I saw that it was quite crowded. There were seven patients already waiting. I joined them in the waiting area. Today the doctor was attending to three patients at a time. Before my turn the compounder came and asked my name and then he retrieved the previous day's slip and put it on the table in front of the doctor. He handed me the thermometer which I immediately put under my arm and waited for him to take the temperature. The doctor called out my name after a few minutes. I went and sat on the patient's chair. I told the doctor, "Doctor Sahib, I did not get any relief from the medicines". The doctor asked, "Is the cough better?" I said, "No, the cough is the same". Then he asked, "Has the fever dowe down?" I said, no. The doctor put the stethoscope on my chest and listened. The compounder informed

him, “She is running 99-degree temperature”. The doctor then shined the torch and examined my throat. After the throat examination, he wrote my name and something else on the slip and handing me the slip, asked me to go to the compounder. I handed my slip to the compounder who then gave it to another compounder who was assembling the medicines. I stood there for some time and then the compounder called my name and told me, “These medicines are for two days as tomorrow the clinic will remain closed. If you do not get any relief by Monday, come back and report”. The compounder took the fee from me which was Rs.100. I left after making the payment.

Visit 3

When I reached the clinic the next day on the third visit, I saw three to four women with small children. I awaited my turn. I heard that all the children had fever, cough and cold. The older people too had come there because they had a cough and fever and sore throat. There were eight patients, before me. When my turn came, the compounder asked me my name and took out my slip and kept it on the Doctor table. He also handed me the thermometer, which I put under my arm and gave it to him after a minute or so. Then my turn came and I said, “Doctor Sahib, I took all the medicines but there was no relief”. The doctor asked, “How is your cough?” I told him, “Doctor Sahib, I have some cough in the day but it is worse in the mornings and night.” The doctor said, “It is cold and because of that you are coughing a lot. If you stay in a warm climate, you won’t have a cough. If the weather improves, your cough will get better too”. Then shining a torch, the doctor checked my throat and used a stethoscope to check my chest. And he was about to check my fever when the compounder said there is no need, I checked the fever. Then the doctor wrote the medicine on the paper and said, “Get this injection, you will feel better quickly”. I said “No” to the injection when the Doctor smiled and said, “Have you taken an oath that you will never take an injection?” I said, “No, but please just give me the medicine; I don’t want to take an injection”. Then the doctor wrote a prescription for a medicine and gave it to the compounder. I got up from the patient’s seat and waited for my medication. The compounder took my name and explained that I should take the medicines for two days, thrice a day with hot water. He said, “And come back after two days and make a report about whether you feel better or not. I asked the compounder what was the fee, so the Compounder asked for a hundred rupees. I gave him the hundred rupees and as I was coming out of the clinic I saw there were about nine patients sitting there waiting for their turn.
